# Pan-cancer analyses identify oncogenic drivers, expression signatures, and therapeutic vulnerabilities in RHO GTPase pathway genes

**DOI:** 10.3389/fbinf.2025.1708800

**Published:** 2025-12-17

**Authors:** Rubén Fernández, L. Francisco Lorenzo-Martín, Víctor Quesada, Xosé R. Bustelo

**Affiliations:** 1 Molecular Mechanisms of Cancer Program, Centro de Investigación del Cáncer, CSIC and Universidad de Salamanca, Salamanca, Spain; 2 Instituto de Biología Molecular y Celular del Cáncer, CSIC and Universidad de Salamanca, Salamanca, Spain; 3 Centro de Investigación Biomédica en Red de Cáncer (CIBERONC), Instituto de Salud Carlos III, Madrid, Spain; 4 Departamento de Bioquímica y Biología Molecular, Universidad de Oviedo, Oviedo, Spain

**Keywords:** RHO GTPases, cancer genomics, signal transduction, tumor suppressors, oncogenes, functional genomics, CRISPR screens, somatic mutations

## Abstract

RHO family GTPases are key regulators of cancer-related processes such as cytoskeletal dynamics and cell migration, proliferation, and survival. Despite this, a comprehensive understanding of RHO signaling alterations across tumors is still lacking. In this study, we present a pan-cancer analysis of 484 genes encoding RHO GTPases, regulators, proximal effectors, distal downstream signaling elements, and components of their proximal interactomes using data from over 10,000 tumor samples and 33 tumor types present in The Cancer Genome Atlas (TCGA). In addition, we have utilized available data from genome-wide functional dependency screens performed in more than 1,000 gene-edited cancer cell lines. This study has uncovered positively selected mutations in both well-known and previously uncharacterized RHO pathway genes. Transcriptomic profiling reveals widespread and tumor-specific differential expression patterns, with some of them correlating with copy number changes. Interestingly, certain regulators exhibit consistent expression profiles across tumors opposite to those predicted from their canonical roles. Co-expression and gene set enrichment analyses highlight coordinated transcriptional programs involving some RHO GTPase pathway genes and their linkage to key cancer hallmarks, including extracellular matrix reorganization, cell motility, cell cycle progression, cell survival, and immune modulation. Functional screens further identify context-specific dependencies on several deregulated RHO GTPase pathway genes. Altogether, this study provides a comprehensive map of RHO GTPase pathway alterations in cancer and identifies new oncogenic drivers, expression-based signatures, and therapeutic vulnerabilities that could guide future mechanistic and translational research in this area.

## Introduction

RHO GTPases can be grouped into the CDC42, RAC, RHOA, RHOD/F, RHOH, RHOU/V, RND, and the most distantly related RHOBTB subfamilies. Most of these GTPases function as molecular switches, cycling between an inactive (GDP-bound) and an active (GTP-bound) state in response to upstream stimuli. Their activity is also modulated by sequestration of the inactive forms in the cytosol. This regulatory cycle is orchestrated by three major protein classes: RHO guanine nucleotide exchange factors (GEFs), which catalyze the activation step using DBL homology (DH), DOCK, or armadillo domains; RHO GTPase-activating proteins (GAPs), which promote the transition of active GTPases back to the off state using the catalytic GAP domain; and RHO GDP dissociation inhibitors (GDIs), which stabilize and sequester inactive RHO GTPases in the cytosol. Once activated, RHO GTPases engage a broad array of proximal effectors that lead to the activation of multibranched signaling cascades that influence the cytoskeletal dynamics, migration, polarity, proliferation, survival, and cell type-specific functions such as lymphocyte development, T-cell activation, neutrophil responses, neurogenesis, and angiogenesis (Bustelo, 2018; Bustelo et al., 2007; Zandvakili et al., 2017).

Given their wide-ranging impact on cellular physiology, most RHO GTPases have long been considered pro-tumorigenic. This view is supported by experimental evidence demonstrating that gain-of-function mutations in some RHO GTPases, RHO GEFs, or downstream effectors can drive or enhance tumorigenesis. Similarly, loss of function of RHO GAPs promotes the same effects (Bustelo, 2018; Zandvakili et al., 2017; Svensmark and Brakebusch, 2019; Porter et al., 2016). Consistent with this idea, mutations in genes encoding RHO GTPase pathway elements have been found in specific cancer types such as diffuse gastric lymphoma, bladder cancer, Burkitt lymphoma, and diffuse large B-cell lymphoma (*RHOA*) (Schaefer et al., 2023; Zhang et al., 2020; Dopeso et al., 2018; Cho et al., 2017; Rohde et al., 2014; Kakiuchi et al., 2014); head and neck squamous cell carcinoma (*RAC1*) (Veeramachaneni et al., 2019; Chang et al., 2016); melanoma (*RAC1* and *PREX2*) (Hodis et al., 2012; Krauthammer et al., 2012; Shain et al., 2015; Lionarons et al., 2019; Berger et al., 2012); and peripheral T-cell lymphoma (*RHOA* and *VAV1*) (Palomero et al., 2014; Sakata-Yanagimoto et al., 2014; Nagata et al., 2016; Zang et al., 2017; Cortes et al., 2018; Abate et al., 2017; Robles-Valero et al., 2021; Robles-Valero et al., 2022) (for reviews, see Bustelo, 2018; Zandvakili et al., 2017; Svensmark and Brakebusch, 2019; Porter et al., 2016). However, recent advances in cancer genomics and functional studies have revealed a more complex and context-dependent landscape for these proteins in cancer. Thus, both gain- and loss-of-function mutations in specific RHO GTPase pathway genes have been detected in human tumors, with some RHO proteins and their signaling components unexpectedly acting as tumor suppressors in certain contexts (e.g., the RHO GEFs VAV1 and TIAM1) (Bustelo, 2018; Zandvakili et al., 2017; Svensmark and Brakebusch, 2019; Porter et al., 2016; Robles-Valero et al., 2017; Hord et al., 1997; Uhlenbrock et al., 2004; Malliri et al., 2006; Yu et al., 2013; Diamantopoulou et al., 2017). Furthermore, genetic and signaling experiments have shown that some RHO signaling elements can influence tumorigenesis through both canonical (GTPase-dependent) and non-canonical (GTPase-independent) mechanisms, adding additional layers of complexity to their biological roles (Bustelo, 2018; Robles-Valero et al., 2017). Notably, mutations in the RHO pathway genes are typically found at low prevalence in cancer genomes (Bustelo, 2018; Zandvakili et al., 2017; Svensmark and Brakebusch, 2019; Porter et al., 2016), suggesting that altered expression levels or aberrant activation by upstream oncogenic signals may constitute their predominant mode of contribution to tumorigenesis. Despite these insights, the prevalence, diversity, and functional consequences of RHO pathway alterations across the landscape of human cancers remain poorly defined. To date, most investigations have focused on individual genes chosen based on historical precedence or laboratory interest rather than by systematic approaches. As a result, a comprehensive, unbiased view of the mutational and transcriptional landscape of RHO GTPases, their regulators, and their downstream effectors across cancer types is still lacking.

To address this knowledge gap, in this study, we conducted a multidimensional analysis of 484 RHO pathway-related genes by integrating data on somatic mutations, gene expression, and copy number variation using the information available from 33 cancer types and >10,000 cancer patients that is publicly available at TCGA (Cancer Genome Atlas Research Network, 2013). In addition, we have used data from screens of functional dependencies across >1,000 CRISPR-Cas9-edited cancer cell lines (Pacini et al., 2021; Tsherniak et al., 2017). Our findings revealed positively selected mutations in specific RHO pathway genes, a highly variegated gene expression pattern for most of them, and the identification of RHO pathway genes that are important to maintain proliferation rates in a widespread or cancer type-specific manner. Together, our study provides a foundational resource for the cancer and RHO GTPase research communities. It also reveals potential targets for further functional studies or therapeutic intervention.

## Results and discussion

### Elaboration of the list of RHO pathway-related genes

To systematically analyze the role of RHO GTPases in cancer, we first compiled a comprehensive list of genes that have been directly or indirectly implicated in RHO GTPase-regulated signaling pathways based on biochemical, signaling, cellular, or large-scale proteomics analyses. Those included all the GTPases, direct regulators, direct effectors, and distal signaling elements according to extensive literature and database searches and proteins that form part of the large-scale RHO interactome according to proximity-dependent biotinylation (BioID)-based proteomics analyses (Bagci et al., 2020). Using these highly inclusive criteria, we ended up with a total of 484 genes encoding the following proteins (Supplementary Table S1): (i) the 23 known members of the RHO subfamily; (ii) the 85 proteins containing DH (2 of them also harboring RHO GAP domains), DOCK, or armadillo domains that are usually involved in promoting RHO GDP/GTP exchange (2 of them also have RHO GAP domains); (iii) the 68 proteins that contain RHO GAP domains that are usually involved in the RHO inactivation step; (iv) the 3 RHO GDIs; (v) the 64 downstream proximal or distal effectors containing kinase domains; and (vi) the 243 downstream proximal or distal signaling elements lacking kinase domains (e.g., regulatory factors associated with upstream receptor signaling, F-actin remodeling, cell migration, or gene transcription). This group of 484 genes will be referred to hereafter as «RHO GTPase pathway genes». It is important to note, however, that many genes selected in these analyses encode proteins that can work both in RHO-dependent and RHO-independent processes (e.g., *PI3KCA*, *PIK3R1*, and those encoding proteins of the large-scale RHO interactome). Due to this, the readers must be aware that any alteration detected in them might be associated with their involvement in RHO-independent cancer programs.

### Somatic mutational landscape of RHO pathway genes across human cancers

We used the whole-exome sequencing data from over 10,000 tumor samples associated with 33 cancer types that were contained in TCGA database to identify the mutational landscape of RHO GTPase pathway genes. This analysis revealed that 152 (31.2%) RHO GTPase pathway genes were mutated in more than 10 cancer types, 208 (42.7%) were mutated in at least two independent cancer types, and 114 (23.4%) were mutated in specific tumor types (Supplementary Figure S1A). Most of these alterations were detected at either low (<3%, 157 genes) or intermediate (3%–10%, 275 genes) prevalence, although a subset of them (42 genes) exhibited mutation rates at frequencies higher than 10% (Supplementary Figure S1A,B). However, since the overall mutation frequency in the foregoing analyses closely reflected the mutational burden found in each analyzed tumor type and the size of the locus involved (Supplementary Figure S1B,C), we further refined these analyses using a random sampling approach to identify the cancer types that displayed higher than expected mutation rates in RHO GTPase pathway genes when tabulated against the overall mutation burden found in the analyzed tumors. Using this approach, we found that mutations in RHO GTPase pathways genes were specifically enriched (*p* < 0.001) in six (18%) TCGA cancer types (BLCA, BRCA, CESC, KIRP, OV, and UCEC) and to a lesser extent (*p* < 0.01) in eight (24%) TCGA cancer types (GBM, HNSC, LAML, LICH, MESO, SARC, THYM, and UCS) (Supplementary Figure S1D; see also Supplementary Table S2 for the abbreviations used for the cancer types compiled in this study). In terms of prevalence, the most relevant cancer type was BRCA (*p* < 0.001), followed by KIRP, THYM, and UCEC (*p* < 0.05) (Supplementary Figure S1E). These findings indicate that 14 (42%) out of the 33 TCGA cancer subclasses harbor statistically significant mutations in at least one RHO GTPase pathway gene (Supplementary Figure S1D,E).

**FIGURE 1:**
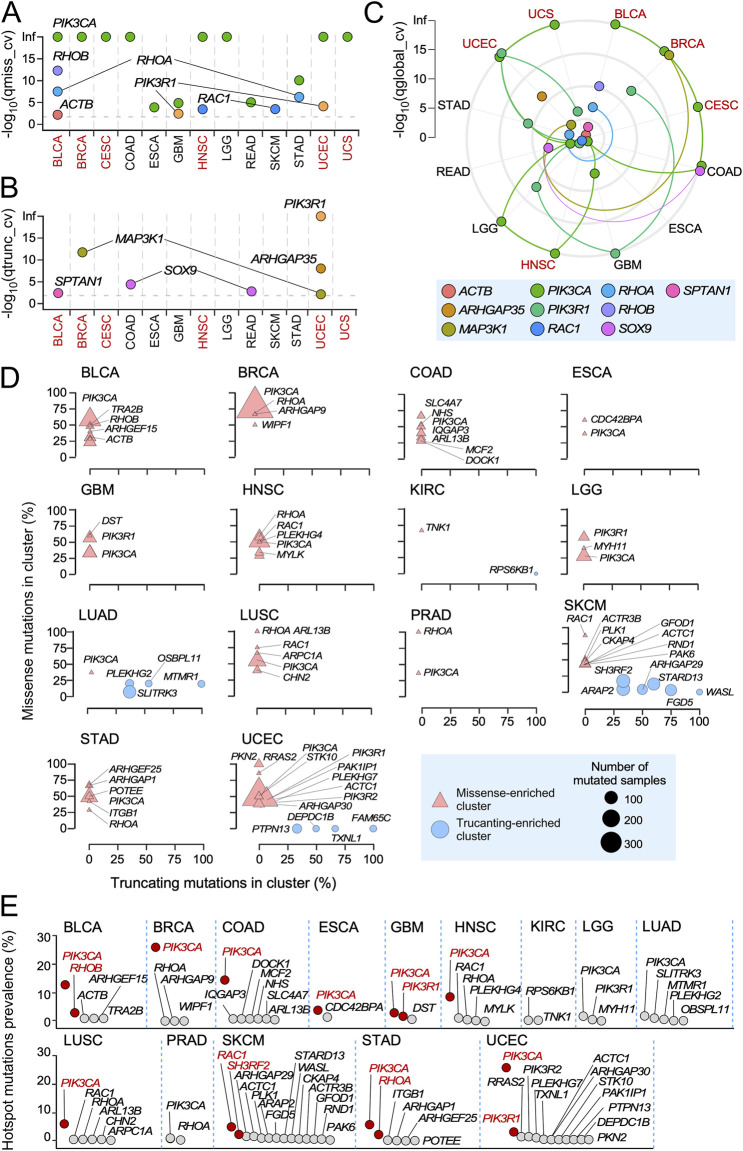
Positively selected and hotspot mutations of RHO pathway genes across TCGA cohorts. **(A,B)** Genes harboring missense **(A)** and truncating **(B)** mutations significantly associated with positive selection signals across TCGA tumors. Tissues enriched in both positively selected and hotspot mutations are shown in red font. **(C)** Distribution of the indicated positively selected mutations (bottom) across TCGA cancer subtypes according to results obtained using the dNdScv method. Each color is associated with one RHO pathway gene harboring somatic mutations under significant positive selection (qglobal_cv value <0.01). Colored lines connect the -log_10_ (qglobal_cv) values associated with each RHO pathway gene through the different TCGA tumors. Tissues enriched in both positively selected and hotspot mutations are shown in red font. **(D)** RHO pathway genes with hotspot mutations. Genes are stratified according to the proportion of missense (y-axis) and truncating (x-axis) mutations present at the identified mutation clusters. Genes with more than 30% of their missense (triangles) or truncating (circles) mutations present at those sites are indicated by their name symbol. The blue box (bottom right) contains further information on the type and size of points used in the representation. **(E)** Prevalence of the identified hotspot mutations in RHO pathway genes. Red dots and font indicate genes with a hotspot mutational prevalence higher than 3% in cancer patients.

We next applied the dNdScv algorithm (Martincorena et al., 2017), which offers a positive selection detection method based on the normalized ratio of non-synonymous to synonymous mutations (dN/dS), to more adequately identify the RHO pathway gene mutations with potential oncogenic driver roles due to the positive or negative selection patterns in specific cancer types. This analysis identified 10 RHO pathway genes (2.1% of the total genes analyzed) with evidence of positive selection (qglobal <0.01) in specific cancer types (Figures 1A–C). Five of these genes, namely, *ACTB* (in BLCA), *RAC1* (in HNSC and SKCM), *RHOA* (in BLCA and STAD), *RHOB* (in BLCA), and *PIK3CA* (in BLCA, BRCA, CESC, COAD, ESCA, GBM, HNSC, LGG, READ, STAD, UCEC, and UCS), harbored positively selected missense mutations (qmis_cv < 0.01, qtrunc_cv > 0.01) (Figure 1A). Apart from *ACTB*, in which mutations have been found in Baraitser–Winter syndrome and coloboma but not in cancer (Johnston et al., 2013; Di Donato et al., 2014), the remaining genes have already been detected as mutated in cancer patients in previous studies (Bustelo, 2018). They all have been linked to pro-tumorigenic functions, except for *RHOB*, in which both tumor suppressor and pro-tumorigenic roles have been assigned (Bustelo, 2018). In addition, 4 out of the 10 genes identified in these analyses contained positively enriched truncating mutations (qmis_cv > 0.01, qtrunc_cv < 0.01): *ARHGAP35* (in UCEC), *MAP3K1* (in BRCA and UCEC), *SPTAN1* (in BLCA), and *SOX9* (in COAD and READ) (Figure 1B). *PIK3R1* harbored both positively selected missense (in GBM and UCEC) and truncating mutations (in UCEC) (Figures 1A,B). All these genes have been associated with either tumor suppressor activities (*ARHGAP35*, *MAP3K1*, and *PIK3R1*) (Bustelo, 2018; Héraud et al., 2019; Cancer Genome Atlas, 2012; Michaut et al., 2016) or with dual, cancer type-specific pro-tumorigenic and tumor suppression functions (*SOX9*) (Panda et al., 2021). Interestingly, the cancer types with positively selected mutations in *RAC1* (HNSC and SKCM) did not overlap with those having positively selected mutations in either *RHOA* (BLCA and STAD) or *RHOB* (BLCA). However, the *RHOA* and *RHOB* genes were both found mutated in BLCA (Figure 1A).

In terms of the total number of positively mutated genes, BLCA (*ACTB*, *PIK3CA*, *RHOA*, *RHOB*, and *SPTAN1*) and UCEC (*ARHGAP35*, *MAP3K1*, *PIK3CA*, and *PIK3R1*) were the most conspicuous (four genes with positively selected mutations each), followed by BRCA (*MAP3K1* and *PIK3CA*), COAD (*PIK3CA* and *SOX9*), GBM (*PIK3CA* and *PIK3R1*), HNSC (*PIK3CA* and *RAC1*), READ (*PIK3CA* and *SOX9*), and STAD (*PIK3CA* and *RHOA*) (two genes with positively selected mutations each). CESC (*PIK3CA*), ESCA (*PIK3CA*), LGG (*PIK3CA*), SKCM (*RAC1*), and USC (*PIK3CA*) each contained only a positively selected gene (Figures 1A–C).

As an alternative avenue to pinpoint mutations in RHO GTPase pathway genes with potentially relevant roles in tumorigenesis, we used the OncodriveCLUSTL algorithm (Arnedo-Pac et al., 2019) to identify hotspot mutations at specific locations in the analyzed genes. We found that 41 RHO GTPase pathway genes contained hotspot missense mutations in BLCA, BRCA, COAD, ESCA, GBM, HNSC, KIRC, LGG, LUAD, LUSC, PRAD, SKCM, STAD, or UCEC (Figure 1D, triangles). A total of 15 out of those 41 genes showed truncation-enriched clusters in a more limited number of cancer types (SKCM, LUAD, UCEC and, to a lesser extent, KIRC) (Figure 1D, circles). RHO pathway genes with the highest percentages of enriched mutations included *PIK3CA* (in 12 cancer types: BLCA, BRCA, COAD, ESCA, GBM, HNSC, LGG, LUAD, LUSC, PRAD, STAD, and UCEC), *RAC1* (in 4 cancer types: BLCA, HNSC, LUSC, and SKCM), *RHOA* (in 4 cancers: BRCA, HNSC, PRAD, and STAD), and *PIK3R1* (in 3 cancers: GBM, LGG, and UCEC) (Figure 1D). In terms of prevalence, the most relevant hotspot mutations were those present in *PIK3CA*, which were found in a large spectrum of cancer types (Figure 1E, red spots). However, as indicated above, the functional impact of these mutations can be broader than the alteration of RHO-dependent pathways, given the implication of PI3K-α in multiple signaling pathways (Cully et al., 2006; Vanhaesebroeck et al., 2010). *RAC1* (SKCM, 4.8%), *RHOB* (BLCA, 2.9%), *PIK3R1* (UCEC, 2.7%; GBM, 2.5%), *RHOA* (STAD, 2.1%), and *SH3RF2* (SKCM, 2%) (Figure 1E, red spots) showed hotspot mutations that were more cancer type-specific. These mutations included RAC1^P29S/L^ and RAC1^E31D^ (Supplementary Figure S2A, top panel); RHOB^P75L/S/T^ (Supplementary Figure S2A, middle panel); and RHOA^Y42S/C^, RHOA^Y34C^, RHOA^F39C^, and RHOA^E40K^ (Supplementary Figure S2A, bottom panel). Hotspot missense (G376R and K379N/E) and truncation (I571Yfs*31, K575Rfs*6, T576Dfs*26, and X582_splice) mutations were also found in the case of *PIK3R1* (Supplementary Figure S2B). Interestingly, the missense and truncation hotspot mutations of this gene were found to be segregated in GBM and UCEC, respectively (Supplementary Figure S2B).

Interestingly, 7 out of the 14 cancer types previously identified as enriched in mutations in RHO GTPase pathway genes did not contain positively selected or hotspot mutations (KIRP, LAML, LIHC, MESO, OV, SARC, and THYM) (Supplementary Figures S1D,E; Figures 1A–C, cancer types in black font; Supplementary Table S3). Conversely, seven cancer types contained positively selected or hotspot mutations that did not have a differential enrichment in mutations for RHO GTPase pathway genes (COAD, ESCA, GBM, LGG, READ, SKCM, and STAD) (Supplementary Figures S1D,E; Figures 1A–C; Supplementary Table S3). In contrast, six cancer types shared both positively selected and hotspot mutations (BLCA, BRCA, CESC, HNSC, UCEC, and UCS) (Supplementary Figure S1D,E; Figures 1A–C, cancer types shown in red font; Supplementary Table S3). We also found RHO GTPase pathway genes with both positively enriched and hotspot mutations in the same cancer types (e.g., *RAC1* in HNSC and SKCM; *RHOA* in STAD; *RHOB* in BLCA; and *PIK3CA* in BLCA, BRCA, ESCA, GBM, HNSC, LGG, STAD, and UCEC; *PIK3R1* in GBM and UCEC) (Figures 1B–D). However, this overlap was not observed in all cases (e.g., *PIK3R1* in LGG, *PIK3CA* in COAD, and *RHOA* in BCLA) (Figures 1B–D). Overall, the hotspot analyses identified four-fold more RHO pathway genes with potential oncogenic driving roles than those based on the detection of positively selected mutations (Figures 1B–D). Interestingly, these analyses also indicated that 13 TCGA cancer types did not contain any positively selected or hotspot mutations according to our statistical criteria (ACC, CHOL, KICH, KIRC, LUAD, LUSC, DLBC, PAAD, PCPG, PRAD, TGCT, THCA, and UVM) (Supplementary Table S3).

### Transcriptional deregulation landscape of RHO pathway genes in human cancers

**FIGURE 2:**
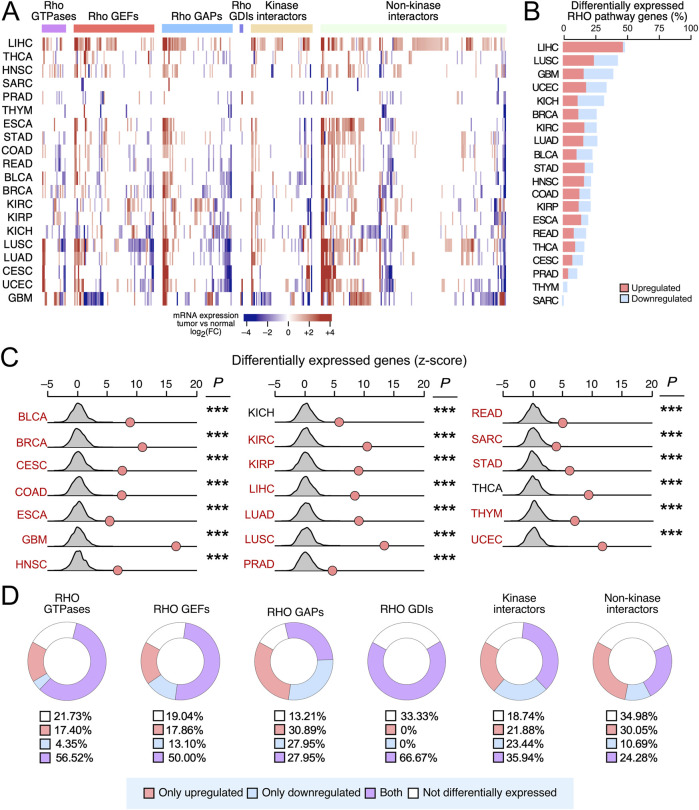
Differential expression of RHO pathway genes in pan-cancer data. **(A)** Heatmap showing the indicated functional categories of RHO pathway genes (top) that are either upregulated (red) or downregulated (blue) in the cancer types indicated on the left compared to their appropriate healthy controls. Expression values are presented as a gradient from the most downregulated (darkest blue) to most upregulated (darkest red) genes according to the scale shown at the bottom. **(B)** Stacked bar diagram representing the percentage (%) of significantly upregulated (red) and downregulated (blue) RHO pathway genes present in the indicated TCGA cancers (left). **(C)** Results from the random sampling analysis of the differential expression of the RHO pathway genes across the indicated TCGA cancers (left), in which we compared the number of differentially expressed RHO pathway genes (red dots) with the expected background distribution of unrelated genes (gray curves). *p-*values were calculated using the Poisson test: ***, *p* < 0.0005. Cancer types with differentially expressed RHO GTPase pathway genes that were also enriched in overall mutations, positively selected mutations, and hotspot mutations are shown in red font. **(D)** Donut charts representing the percentage of RHO pathway genes of the indicated functional categories (top) that show the indicated pattern of differential expression according to our pan-cancer analyses (see blue box at the bottom) The percentage (%) of each expression pattern (blue box) is also indicated.

We next explored the degree of deregulation of RHO pathway genes at the mRNA level. To this end, we performed gene expression analyses across 20 TCGA cancer cohorts that included matched normal tissue controls. Overall, we found that 358 (74%) of the 484 analyzed RHO pathway genes were deregulated in a statistically significant manner in at least one cancer type (Figures 2A,B). The extent and type of deregulation of the transcripts, however, varied substantially across the different tumor types (Figures 2A,B). For example, >40% of RHO pathway genes were differentially expressed in LIHC, LUSC, and GBM, while <3% were found in SARC and THYM (Figure 2B). In addition, we found that upregulation events were highly predominant in LIHC, whereas in other cases, downregulation events prevailed (GBM, KIH, PRAD, THYM, and SARC) (Figure 2B). Random sampling analyses confirmed that these alterations occurred at significantly higher rates than expected by chance in all 20 analyzed TCGA cancer types (Figure 2C), suggesting that transcriptional deregulation of RHO signaling is a recurrent and biologically relevant feature of tumorigenesis. Cancer types with differentially expressed RHO GTPase pathway genes included those that were also enriched in overall mutations, positively selected mutations, and hotspot mutations (Figure 2C), along with two TCGA cancer types (KICH and THCA) that were not identified in our previous mutational analyses (Figure 2C). The transcripts for RHO GAPs were the most frequently deregulated (86.8%), followed by downstream kinases (81.3%), RHO GEFs (81.0%), RHO GTPases (78.3%), RHO GDIs (66.7%), and downstream elements lacking kinase activity (65.0%) (Figure 2D). However, a minority of RHO GTPase pathway genes were consistently upregulated (Figure 2D, red) or downregulated (Figure 2D, blue) in all cancer types. RHO GAP-encoding genes were the RHO GTPase pathway genes with more consistent patterns of pan-cancer upregulation or downregulation by far (58.8% of the analyzed RHO GAPs), followed by kinase interactors (45.3%), non-kinase interactors (40.7%), RHO GEFs (31.0%), RHO GTPases (21.8%), and RHO GDIs (0%) (Figure 2D).

**FIGURE 3:**
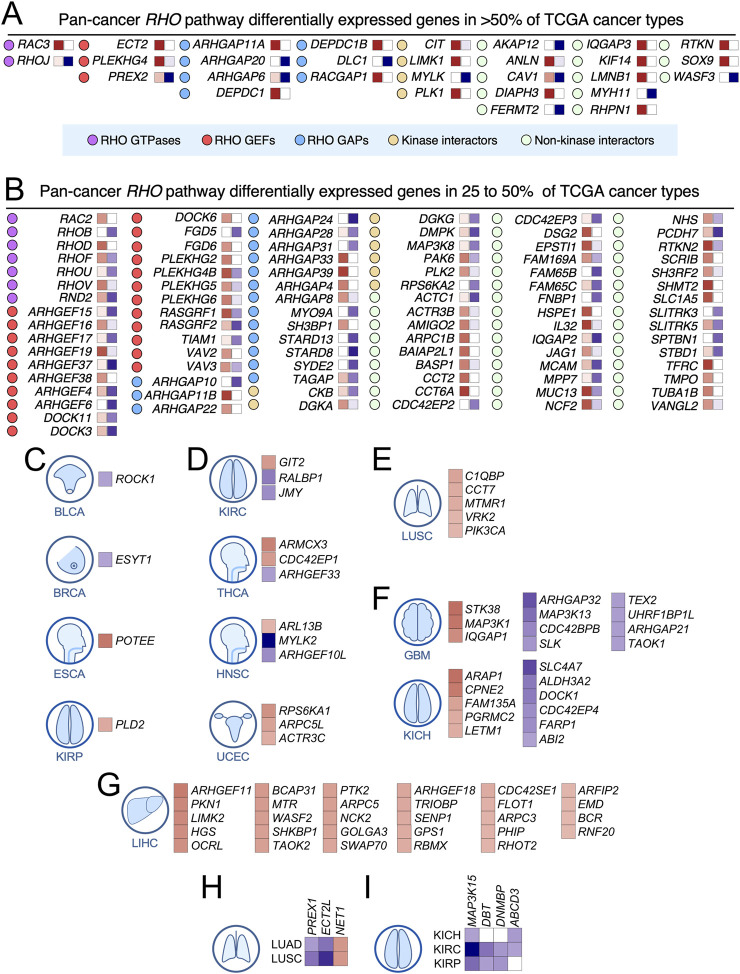
Differential expression of RHO pathway genes in pan-cancer data. **(A,B)** RHO pathway genes of the indicated functional categories (see blue box at the bottom) that are significantly upregulated (red boxes on the right of each gene) or downregulated (blue boxes in the right of each gene) in more than 50% **(A)** or 25% **(B)** of the analyzed TCGA cohorts. The intensity of each colored square is directly proportional to the total number of tumors in which each gene is significantly upregulated or downregulated. Associated cancers in which the red squares are darker than the blue squares were considered pan-cancer upregulated RHO pathway genes. Those with blue squares darker than the red squares were considered pan-cancer downregulated RHO pathway genes. **(C–I)** Examples of RHO pathway genes with differential expression patterns limited to one **(C–G)**, two **(H)**, or three **(I)** cancer types. For the expression color codes, see panels A and B.

In terms of penetrance, we found that 44% of deregulated RHO pathway genes were differentially expressed in more than five TCGA cancer types and defined this subset as «pan-cancer differentially expressed» RHO GTPase pathway genes (Figures 3A,B). Other genes showed more limited spectra of differential expression patterns across cancer types, including some in which deregulated expression was only found in one (20%), two (14%), three (14%), or four (8%) cancer types (see examples in Figures 3C–I). Consistently upregulated transcripts encoding RHO GTPase pathway elements across 25%–50% (not underlined in the text) and >50% (underlined genes) in the 20 analyzed TCGA cancer types included 4 RHO GTPases (*RAC3*, *RAC2*, *RHOD*, and *RHOV*), RHO GEFs (*ECT2*, *PLEKHG4*, *ARHGEF16*, *ARHGEF19*, *ARHGEF38*, *DOCK6*, *FGD6*, *PLEKHG2*, and *VAV2*), 12 RHO GAPs (*ARHGAP11A*, *DEPC1*, *DEPC1B*, *RACGAP1*, *ARHGAP11B*, *ARHGAP11B*, *ARHGAP22*, *ARHGAP33*, *ARHGAP39*, *ARHGAP4*, *ARHGAP8*, and *SH3BP1*), 4 downstream kinases (*CIT*, *LIMK1*, *PLK1*, and *PLK2*), and 24 non-kinase downstream elements (*ANLN*, *DIAPH3*, *IQGAP3*, *KIF14*, *LMNB1*, *RHPN1*, *RTKN*, *SOX9*, *ACTR3B*, *AMIGO2*, *ARPC1B*, *BAIP2L1*, *CCT2*, *CCT6A*, *DSG2*, *EPSTI1*, *HSPE1*, *SCRIB*, *SHMT2*, *SLC1A5*, *TFRC*, *TMPO*, *TUBA1B*, and *VANGL2*) (Figures 3A,B). Consistently downregulated transcripts across >50% (underlined in the text) and 25%–50% (not underlined in the text) of the analyzed cancer types included 2 RHO GTPases (*RHOJ*, *RHOB*, and *RHOU*), 4 RHO GEFs (*ARHGEF15*, *ARHGEF17*, *ARHGEF6*, and *FGD5*), 10 RHO GAPs (*ARHGAP20*, *DLC1*, *ARHGAP10*, *ARHGAP24*, *ARHGAP28*, *ARHGAP31*, *MYO9A*, *STARD8*, *STARD13*, and *SYDE2*), 2 downstream kinases (*MYLK* and *RPS6KA2*), and 9 non-kinase downstream elements (*AKAP12*, *FERMT2*, *MYH11*, *WASF3*, *CDC42EP2*, *FAM65B*, *FNBP1*, *SLITRK3*, and *SPTBN1*) (Figures 3A,B). These results suggest that these consistent expression patterns across cancers might have functional relevance. In line with this, we found that five of those upregulated genes (*DEPC1B*, *IQGAP3*, *PLEKHG2*, *PLK1*, and *SOX9*) and six of those downregulated genes (*FGD5*, *MYLK*, *MYH11*, *RHOB*, *STARD13*, and *SLITRK3*) also harbored positively selected or hotspot mutations according to previous analyses (see above, Figure 1). However, many RHO GTPase pathway genes showed different patterns of expression (up- and downregulation) depending on the cancer type analyzed (Figures 2C, 3A–H). This is the case, for example, for 2 RHO GTPase-encoding genes (*RHOF* and *RND2*) (Figure 3B), 9 RHO GEF-encoding genes (*PREX2*, *ARHGEF4*, *DOCK3*, *DOCK11*, *PLEKHG4B*, *PLEKHG5*, *RASGRF1*, *RASGRF2*, and *VAV3*) (Figures 3A,B), 2 RHO GAP-encoding genes (*ARHGAP6* and *TGAP*) (Figures 3A,B), 5 kinase-encoding genes (*CKB*, *DGKA*, *DGKG*, *DMPK*, and *PAK6*) (Figure 3B), and 10 genes encoding either non-kinase downstream or interactome elements (*CAV1*, *FAM169A*, *FAM15C*, *IQGAP2*, *MCAM*, *MUC13*, *NCF2*, *NHS*, *PCDH7*, and *RNKN2*) (Figures 3A,B). This cancer type-specific expression pattern could represent bystander events without any functional relevance or the fact that some of those genes encode proteins with dual functions depending on the cancer context. Alternatively, it might reflect the enrichment of a specific cell population that originates the cancer and has expression levels of RHO pathway genes different from those of the remaining cell types that form part of the tissue from which the cancer originated.

These analyses also indicated that many RHO GTPase pathway genes did not follow the expected transcriptional deregulation patterns according to their canonical functions (Figures 2C, 3; Supplementary Figure S3). For example, RHO GEFs, which act as positive regulators of RHO GTPases, were expected to be upregulated in most tumor types. However, this expected expression pattern was only clearly observed in some of them (*ARHGEF11*, *ARHGEF18*, *ARHGEF19*, *DOCK6*, *ECT2*, *FGD6*, *NET1*, *PLEKHG2*, and *VAV2*) (Figures 3A,B,G,H; Supplementary Figure S3). Pro-tumorigenic functions for some of those RHO GEFs (*ARHGEF11*, *ARHGEF18*, *ARHGEF19*, *ECT2*, *PLEKHG2*, and *VAV2*) have been described in some tumor models in previous studies (Bustelo, 2018; Du et al., 2020; Citter et al., 2012; Menacho-Marquez et al., 2013; Lorenzo-Martín et al., 2020a; Lorenzo-Martín et al., 2020b; Rodríguez-Fdez et al., 2020; Lorenzo-Martín et al., 2022). Likewise, RHO GAPs, which act as negative regulators of RHO GTPases, were supposed to be downregulated in most tumor types. However, such a pattern was only found in the cases of *ARHGAP20*, *ARHGAP21*, *ARHGAP24*, *ARHGAP31*, *ARHGAP32, DLC1*, *MYOA9*, *STARD8*, *STARD13*, and *SYDE1* (Figures 3A,B,F; Supplementary Figure S3). Tumor suppressor-like functions for some of those RHO GAPs have been described before (*ARHGAP20*, *ARHGAP24*, *DLC1*, *MYOA9*, *STARD8*, and *STARD13*) (Bustelo, 2018; Liu et al., 2021; Xu et al., 2016; Yang et al., 2022; Ren and Li, 2021; Yuan et al., 1998; Liu et al., 2024; Durkin et al., 2007; Ching et al., 2003). In contrast, we found a subset of RHO GEFs (*ARHGEF4*, *ARHGEF6*, *ARHGEF15*, *ARHGEF17*, *ARHGEF37*, *FGD5*, *PREX2*, *TIAM1*, and *RASGRF2*) and RHO GAPs (*ARHGAP4*, *ARHGAP8*, *ARHGAP11A*, *ARHGAP11B*, *ARHGAP22*, *ARHGAP33, ARHGAP39*, *DEPC1*, *DEPC1B*, *RACGAP1*, and *SH3BP1*) that were downregulated and upregulated in the analyzed cancer cohorts, respectively (Figures 2D, 3A,B,D,F,H; Supplementary Figure S3). Cases of the opposite spectra of mRNA expression have been also found for the remaining functional subclasses of RHO GTPase pathway genes studied in this work (Figures 2D, 3A,B; Supplementary Figure S3). This unexpected pattern of expression can be due to several causes. First, it is possible that the encoded proteins can play roles in cancer that do not follow their expected canonical functions. Such functions can be catalytically dependent or independent, as reported before (Bustelo, 2018). Alternatively, it is possible that the overexpression of a given RHO GTPase pathway element could reflect its relevance in essential processes, such as cell division. This is the case, for example, of the key role played by RACGAP1 in cytokinesis and other pro-tumorigenic pathways (Lores et al., 2014; Lin et al., 2024). Consistent with this idea, we have found that three of the upregulated RHO GAP-encoding genes identified in our *in silico* analyses (*ARHGAP11A*, *ARHGAP11B*, and *DEPDC1*; Figures 3A,B) do perform RACGAP1-like functions in cytokinesis (unpublished data). Further supporting this hypothesis, previous studies have also demonstrated pro-tumorigenic roles for two of the RHO GAPs found to be upregulated in our analyses (*ARHGAP11A* and *RACGAP1*) (Lawson et al., 2016). Alternatively, the non-canonical deregulation of the expression levels of some RHO GTPase pathway genes might indicate that such changes represent just a bystander, functionally irrelevant event (due to being part of larger transcriptional programs or the expansion of the cell type that originates the cancer, where it is found to be deregulated). Future wet-laboratory analyses are required to decipher these issues.

To further explore the biological basis of the differential expression patterns of RHO GTPase pathway genes, we investigated whether such an expression was associated with catalytic specificity toward their GTPase substrates. To this end, we used the proposed list of substrates (CDC42, RAC1, and RHOA) for these RHO GTPase regulators, previously generated based on information gathered from fluorescence resonance energy transfer-based activity assays (Muller et al., 2020). Among the differentially expressed RHO GEFs, we found that the upregulated subset primarily contained RHOA- (50%) and CDC42-specific (18.2%) GEFs (Supplementary Figure S4A). In contrast, the downregulated subset mostly contained (50% of cases) RAC1-specific GEFs (Supplementary Figure S4A). However, these data might not reflect changes associated with a functional trend since the human genome contains an approximately 7:1 ratio between RHOA and RAC1-specific GEFs (Muller et al., 2020). Among the 12 identified pan-cancer downregulated RHO GAPs, we found that 50% of them were specific for RHOA, while only 1 (*SYDE2*) was considered RAC1-specific. In contrast, 36% of the 11 upregulated RHO GAPs were specific for RAC1, while only 2 (*ARHGAP8* and *ARHGAP11B*) were proposed to be RHOA-specific (Supplementary Figure S4B). Given that there is a similar number of RHOA- and RAC1-specific RHO GAPs encoded in the human genome (Muller et al., 2020), the percentages obtained in this case suggest that many cancer types favor the activation of RHOA and the inactivation of RAC1 through the downregulation and upregulation of RHOA GAPs and RAC1 GAPs, respectively.

### Impact of the copy number variations on the expression of RHO pathway genes

**FIGURE 4:**
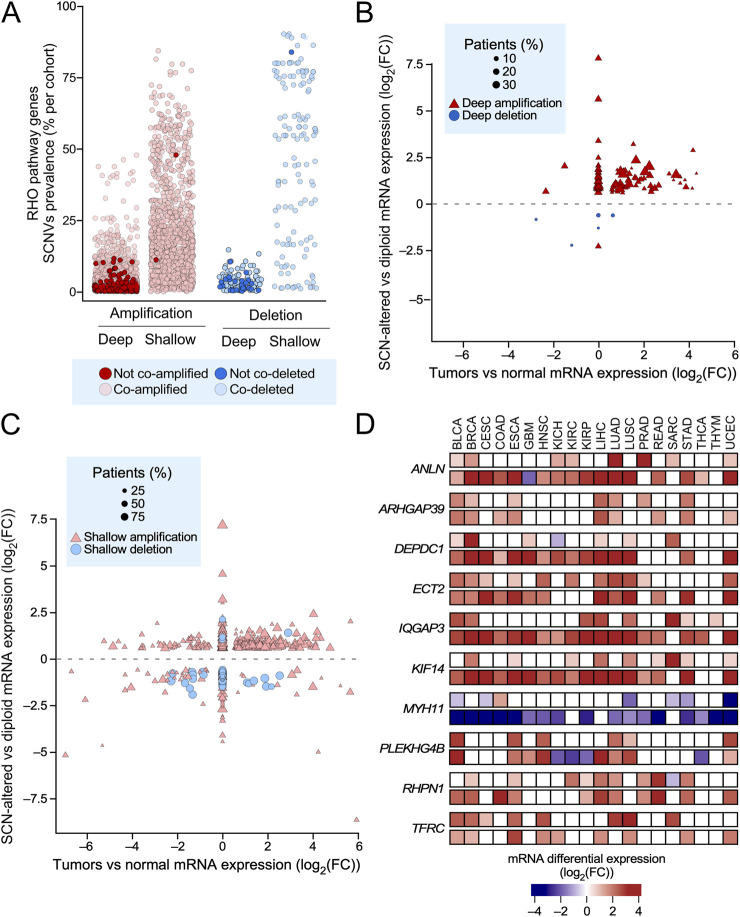
Somatic copy number variations associated with transcriptional deregulation of RHO pathway genes in TCGA tumors. **(A)** SCNVs associated with RHO pathway genes found in pan-cancer data. The dot colors represent copy number gains (red) and losses (blue). Darker red and blue colors represent copy number variations of RHO pathway genes that are unrelated with the concurrent amplification or deletion of cancer drivers present in the COSMIC database. See blue box at the bottom for further information. **(B,C)** Transcriptional status analysis of RHO pathway genes whose differential expression correlates with highly deep **(B)** or shallow **(C)** SCNVs (>10% of the patients). In B and C, the y-axes represent the log_2_(FC) expression value of the RHO pathway genes calculated by comparing the mRNA levels between SCN-positive and diploid cancer samples. The x-axes represent the log_2_(FC) expression value of RHO pathway genes obtained by comparing the mRNA levels between cancer and healthy samples. See inset (blue box) for further information about the symbols used in these graphs. **(D)** Heatmap showing the log_2_(FC) expression values associated with the differential expression of 10 pan-cancer deregulated RHO pathway genes that were calculated by comparing the mRNA levels in TCGA cancers between: (a) SCN-positive and diploid cancer samples (top lanes) and (b) cancer and normal samples (bottom lanes). Expression values are presented as a gradient from the most downregulated (darkest blue) to the most upregulated (darkest red) genes according to the scale shown at the bottom.

Somatic copy number variations (SCNVs) can also contribute to changes in gene expression. To explore this possibility, we used a new algorithm developed by us (CiberAMP) that can establish direct correlations between gene copy number changes and significant shifts in the mRNA expression levels (Caloto et al., 2022). As a first step, we found using CiberAMP that 87% of the 484 RHO GTPase pathway genes undergo SCNV events in at least one of the 33 analyzed TCGA cancer types. When focusing on SCNVs with high prevalence (affecting >10% of patients), this proportion decreases to 74% (Figure 4A). Shallow amplifications were the most frequent SCNVs across TCGA tumors, followed by deep amplifications and shallow and deep deletions (Figure 4A). To distinguish driver events from passenger alterations co-occurring with known oncogenes, we used CiberAMP to identify the SCNVs in RHO pathway genes that mapped in close vicinity with well-established oncogenes listed in the COSMIC Cancer Gene Census (CGC). We found that 97% of SCNVs associated with RHO GTPase pathway genes significantly co-occurred with amplifications or deletions of CGC oncogenes (Figure 4A, light-colored circles), indicating that most SCNVs detected for the RHO GTPase pathway genes are probably mere passenger events in these tumors. In line with this, we found using CiberAMP that 70% of all the RHO GTPases undergoing deep (Figure 4B) or shallow (Figure 4C) SCNVs did not change their expression according to the gene loss or gene amplification event detected. However, we identified 10 pan-regulated RHO pathway genes in which the gene losses (*MYH11*) or gene amplifications (*ANLN*, *ARHGAP39*, *DEPDC1*, *ECT2*, *IQGAP3*, *KIF14*, *PLEKHG4B*, *RHPN1*, and *TFRC*) reinforced the transcriptional changes found in analyses performed using diploid cancer samples and healthy controls (Figure 4D). Two of those genes also undergo hotspot mutations (*MYH11* and *IQGAP3*) according to our prior analyses (Figure 1D). Although small, changes in the expression of these genes must be relevant, given that many of them have been linked to either tumor-suppressing (*MYH11*) or tumor-promoting (remaining) functions (see also below, Figure 6B). Taken together, these data indicate that only a minority of RHO GTPase pathway genes appear to change their expression because of SCNVs.

### Coordinated pan-cancer deregulation of RHO-specific GEFs and GAPs

**FIGURE 5:**
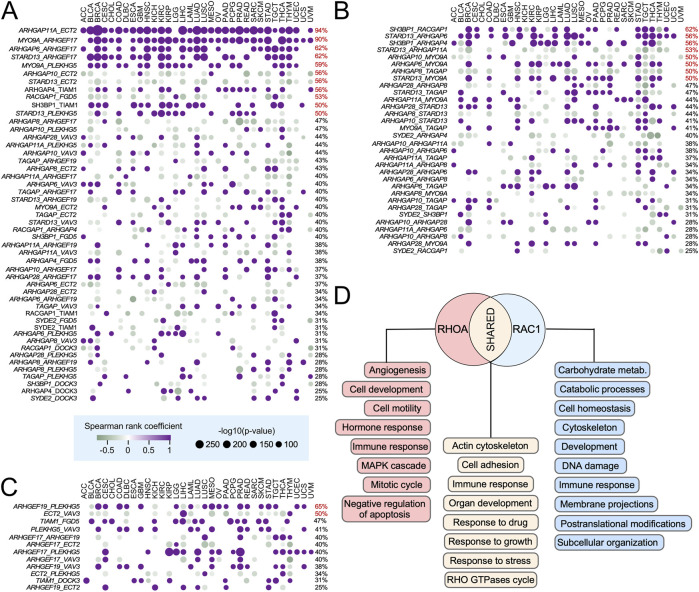
Ontological analysis of pan-cancer deregulated RHO GEFs and GAPs. **(A–C)** Co-expression analysis of indicated pairs of RHO GAPs–RHO GEFs (**A**, left), RHO GAPs (**B**, left), and RHO GEFs (**C**, left) in indicated tissues (**A–C**; top). The dot size and color of each analyzed pair are associated with –log_10_(*p*) and Spearman correlation coefficient (positive correlations in purple and negative correlations in green), respectively. The percentage of TCGA cancers in which the expression of the indicated pair of genes is significantly co-regulated is indicated on the right. Co-expressed pairs that are detected in more than 50% of the cancer types analyzed are shown in red. **(D)** Top global gene ontology (GO) terms associated with RHOA- and RAC1-specific pan-cancer deregulated GEFs and GAPs across the TCGA tumor types. Functions for RHOA- and RAC1-specific GEFs are shown in red and blue boxes, respectively. Functional categories shared by RHOA- and RAC1-specific GEFs are shown in light brown boxes.

Given our observation that many RHO GEFs and GAPs are consistently deregulated across multiple tumor types (Figures 3A,B), we next explored whether such expression patterns could be associated with the activation of large-scale transcriptional programs in specific cancer types. To assess this issue, we first performed co-expression analyses between all possible pairs of RHO GEFs, RHO GAPs, and RHO GEFs–RHO GAPs across the 33 TCGA cohorts to identify coregulatory events. These analyses revealed that 98% of all possible RHO GEF–RHO GAP gene pairs show significant co-expression in >25% of the analyzed tumor types (Figures 5A,B). The most relevant co-regulated GAP–GEF pairs (>50% of cancer types) included *ARHGAP11A–ECT2*, *MYO9A* with *ARHGEF17* and *PLEKHG5*, *ARHGAP6*–*ARHGEF17*, *SH3BP1*–*TIAM1*, and *STARD13*–*PLEKHG5* (Figure 5A, purple dots), while the most relevant anti-regulated GAP–GEF pairs included those of *ECT2* with *ARHGAP10* and *STARD13* and *RACGAP1–FGD5* (Figure 5A, green dots). We also found cases of strong co-regulation among RHO GAP pairs in pan-cancer data, including *SH3BP1* with *RACGAP1* and *ARHGAP6*; *STARD13* with *ARHGAP6*, *ARHGAP11A*, and *MYO9A*; and *ARHGAP10–MYO9A* (Figure 5B, purple dots), along with cases of strong anti-regulation (*STARD13–ARHGAP11A*, *ARHGAP10–MYOA9*, and *ARHGAP8–TGAP*) (Figure 5B). In contrast, the pattern of strong co-regulation (*ARHGEF19–PLEHHG5*) and anti-regulation (*ECT2–VAV3*) among RHO GEFs was much more limited (Figure 5C). Additional pairs with lower percentages of distribution in cancer types (less than 50%) were found for all those categories (Figures 5A–C).

This pattern of co-regulated expression suggested that the transcriptional alterations found in pan-cancer differentially expressed RHO GTPase pathway genes could be the consequence of their implication in broader, pan-cancer regulatory gene expression networks. To further explore this possibility, we next expanded the list of co-regulated genes by identifying, in an unbiased manner, the genes of the human genome that were either positively or negatively co-regulated with the differentially expressed pan-cancer RHO GEFs and GAPs. We then used this information to build RHO GTPase-centered, weighted gene lists to carry out gene set enrichment analysis (GSEA) using Gene Ontology (GO) biological processes. The top-100 GO terms per group were ranked by the median normalized enrichment score (NES) across the tumor types. For RHOA-specific networks, enriched processes included tumor angiogenesis, MAPK signaling, inhibition of apoptosis, tumor growth, and cell migration (Figure 5D, red boxes). For RAC1-specific networks, the enriched processes were associated with transcriptional programs involved in membrane projection dynamics, DNA damage responses, and metabolic regulation (Figure 5D, blue boxes). Importantly, 50% of the top GO terms were shared between the RHOA- and RAC1-specific networks (Figure 5D, brown boxes). Together, these data indicate that many cancers engage a coordinated, pan-cancer transcriptional program that includes the modulation of RHO GEF- and RHO GAP-encoding genes. However, we cannot exclude the possibility that some of these co-regulatory events could result from feedback or compensatory responses aimed at maintaining homeostasis in RHO-regulated pathways.

### Functional impact of the elimination of RHO pathway genes in cancer cell lines

**FIGURE 6:**
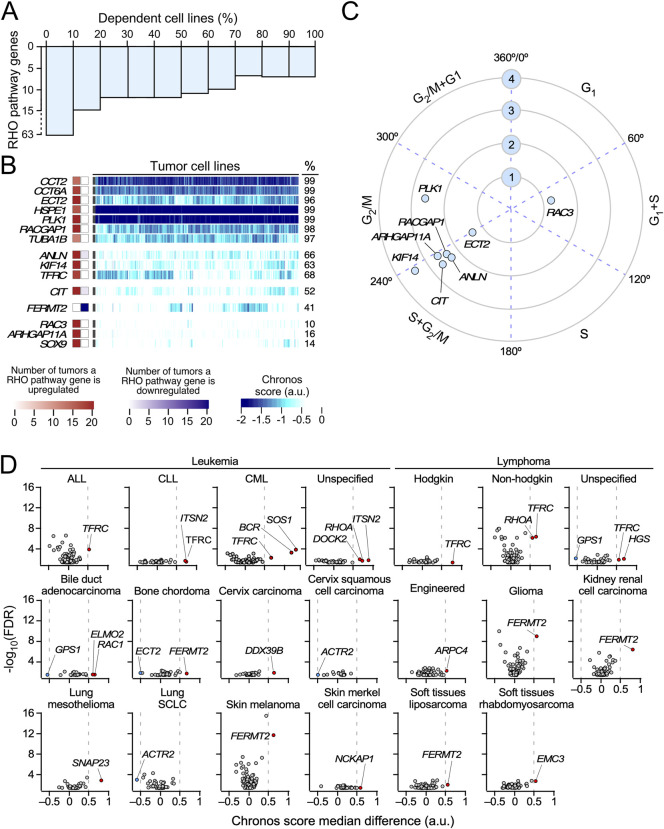
Cancer cell line dependence on the expression of pan-cancer deregulated RHO pathway genes. **(A)** Bar plot representing the percentage of cancer cell lines associated with a dependency Chronos score < −0.5 when a pan-cancer deregulated RHO pathway gene is knocked-out. **(B)** Heatmap showing the Chronos scores obtained for the pan-cancer deregulated RHO pathway genes in the 1,233 cancer cell lines analyzed in this study. Only genes associated with Chronos scores < −0.5 in more than 10% of the analyzed cell lines are represented. Squares on the left show the level of differential expression found for each of those genes across TCGA cohorts (red, upregulated; blue, downregulated). Values are represented as color gradients, as indicated in the scales shown at the bottom of the figure. Values on the right provide the percentage (%) of cell lines that are dependent on the indicated genes. **(C)** Polar coordinates for pan-cancer RHO pathway genes identified in panel B that were found to significantly oscillate in synchrony with the cell cycle (FDR ≤0.001, logCPM ≥1, FC ≥ 1.5) in cell-sorted HeLa and U2OS cells. The numbered blue circles indicate the expression level (log_2_(FC)) of each transcript in the indicated cell cycle stages. **(D)** Vulnerabilities of cancer cell lines representative of the indicated cancer types (top) to the depletion of the expression of RHO pathway genes. The y-axis values represent the -log_10_(FDR-adjusted *p-*value) associated with each gene when comparing the distribution of Chronos scores between a subgroup of tumor cells from the same subtype with another subgroup gathering all the remaining cell lines from different subtypes in the study. The x-axis values represent the difference in Chronos scores for each gene between the two subgroups. Genes associated with a |median difference| > 0.5 and a *p* < 0.01 are considered to exert a significant impact on the proliferation of cancer cell lines from a particular tumor subtype. Transcripts whose knockouts reduce and increase the proliferation of the indicated cancer cell types are shown as red and blue dots, respectively. ALL, acute lymphocytic leukemia; CLL, chronic lymphocytic leukemia; CML, chronic myelogenous leukemia; Engineered, genetically engineered cancer cell lines; SCLC, small-cell lung cancer.

To investigate whether the depletion of the RHO pathway genes confers a proliferative disadvantage to tumor cells, we next assessed the dependency of 1,032 cancer cell lines on the expression of RHO pathway genes using data from genome-wide CRISPR/Cas9 essentiality screens (Pacini et al., 2021; Tsherniak et al., 2017). Among the 129 pan-cancer deregulated RHO pathway genes identified in TCGA tumors (Figure 3), the depletion of 63 genes significantly reduced proliferation in at least one cancer cell line (Chronos score < −0.5) (Figure 6A). Notably, 15 of these genes were required for the growth of more than 10% of all the tested cell lines, indicating a broad spectrum of dependency (*CCT2*, *CCT6A*, *ECT2*, *HSP1*, *PLK1*, *RACGAP1*, *TUBA1B*, *ANLN*, *KIF14*, *TFRC*, *CIT*, *FERMT2*, *RAC3*, *ARHGAP11A*, and *SOX9*) (Figure 6B). Of these 15 genes, 4 were also detected in our SCNV studies (*ANLN*, *ECT2*, *KIF14*, and *TFRC*) (Figure 4D). The expression of 8 of these 15 genes was also strongly associated with cell cycle progression, namely, during the G_1_ and S (*RAC3*), S and G_2_/M (*ANLN*, *ARHGAP11A*, *CIT*, *ECT2*, and *KIF14, RACGAP1*), and G_2_ to M (*PLK1*) phases (Figure 6C). This suggests that the broad impact of the inactivation of these eight genes on the proliferation of cancer cell lines is due to their specific roles in cell cycle progression or cytokinesis.

We next assessed whether RHO pathway gene dependencies vary in a tumor type-specific manner. To this end, we stratified cell lines based on the tissue of origin and compared the distribution of the Chronos scores for each RHO pathway gene within each tumor group against all other cancer types combined. This analysis produced two values per gene–cancer type pair: (i) the difference in the median Chronos scores and (ii) a corresponding false discovery rate (FDR)-adjusted *p-*value from a two-group comparison. Genes showing a median difference >0.5 and FDR-adjusted *p* < 0.01 were considered to exhibit significant tumor-specific vulnerability. This approach revealed multiple lineage-specific dependencies. In hematopoietic malignancies, *TFRC*, a pan-cancer upregulated RHO pathway gene (Figure 3B), was essential for the proliferation of all leukemia and lymphoma-derived lines (Figure 6D). Chronic lymphocytic leukemia (CLL) cells showed dependency on *ITSN2* (Figure 6D); chronic myeloid leukemia (CML) cell lines showed dependency on *SOS1* and *BCR*; and non-Hodgkin lymphomas showed dependency on *RHOA* (Figure 6D). The role of BCR in these cells must be due to the down-modulation of the oncogenic BCR–ABL1 fusion protein that is typical of this tumor type rather than due to the intrinsic function of BCR. Unspecified leukemia cell lines were dependent on *DOCK2*, *ITSN2*, and *RHOA*, whereas unspecified lymphoma cells relied on *HGS* and *GPS1* (Figure 6D). In solid tumors, *FERMT2* was important for the proliferation of cell lines from bone chordoma (together with *ECT2*), glioma, kidney renal cell carcinoma, skin melanoma, and soft tissue sarcoma (Figure 6D). *ELMO2*, *RAC1*, and *GPS1* were important for bile duct adenocarcinoma; *DDX39B* was important for cervix carcinoma; *ACTR2* was important for both cervix squamous cell carcinoma and small-cell lung cancer; *SNAP23* was important for lung mesothelioma; *NCKAP1* was important for skin Merkel cell carcinoma; and *EMC3* was important for soft tissue rhabdomyosarcoma (Figure 6D). Interestingly, we observed that the depletion of three of the differentially expressed RHO pathway genes (*ACTR2*, *ECT2*, and *GPS1*) led to higher proliferation rates in some cell lines (Figure 6D). Together, these analyses underscore the functional relevance of the deregulation of some RHO GTPase pathway genes for the potential fitness of specific cell lines and, possibly, cancer types.

## Concluding remarks

In this study, we integrated genomic, transcriptomic, and functional dependency data from large-scale public resources to generate the first comprehensive dissection of alterations found in the RHO GTPase signaling landscape across human cancers. As inferred from previous analyses (Bustelo, 2018; Zandvakili et al., 2017; Svensmark and Brakebusch, 2019), our findings indicate that the contribution of genetic alterations (mutations and SCNVs) is rather limited. Despite this, we have found a small but significant number of genes that contain positively selected mutations, hotspot mutations, and SCNVs in specific TCGA cancer subtypes (Supplementary Table S3). Additional genes, including some of the aforementioned category, also undergo changes in expression (Supplementary Table S3), further indicating that such alterations might have functional relevance.

Our results also indicate that, as anticipated (Bustelo, 2018; Zandvakili et al., 2017; Svensmark and Brakebusch, 2019), the most common deregulation found for RHO GTPase pathway genes is by far the type that is associated with changes in transcript expression. Our study also suggests that the deregulation of the expression of most RHO GTPase pathway genes cannot be interpreted as the consequence of just their intrinsic or individual functional impact on cancer cells; rather, they are the components of large transcriptional programs that become activated in cancer cells (Figure 5). Thus, rather than being drivers *per se*, it is likely that these proteins will mostly work as ancillary factors that participate in large-scale biological processes regulated by yet unidentified transcriptional factors. Nevertheless, we found a minority of genes whose expression is key to the proliferation of specific cancer cell lines according to the analyses performed using data from CRISPR–Cas9 gene-editing-based screenings.

Although our study provides a comprehensive analysis to date of RHO signaling dysregulation in cancer, the readers must be aware that several limitations remain. First, the functional impact of many identified mutations (especially those that are not positively selected or not found in hotspots) and differential expression patterns found across cancers in our work remains to be experimentally validated. Second, it must be underscored that the deregulation observed at the transcript level does not necessarily match the expression or activity levels of the encoded proteins. Third, changes in mRNA and protein expression might not always correlate with a functional impact at the cellular level due to the activation of buffering signaling events. Likewise, they can simply reflect the enrichment of cancer cells that “inherit” the expression landscape of the specific cell type that originated the cancer. Fourth, our analyses will not identify genes encoding signaling components of RHO GTPase-regulated pathways whose activity is modulated by upstream oncogenic signals rather than by mutations or gene expression changes. Finally, the functional screenings may yield incorrect information in the case of sgRNAs that do not properly inactivate the expected target gene. To overcome these problems, our studies must be complemented with additional phosphoproteomic, proteomic, and activity-based profiling analysis in the future to adequately identify the full spectrum of mechanisms by which RHO GTPase-regulated pathways impact cancer cell biology and pathophysiology. Regardless of these caveats, our study has generated a comprehensive catalog of genetic and non-genetic mechanisms affecting RHO GTPase pathway genes in cancer. This roadmap will be relevant to guide future efforts aimed at targeting RHO GTPase-regulated pathways in oncological malignancies.

## Experimental procedures

### Curation of a list of genes involved in the RHO GTPase pathways

To compile a list of genes involved in the RHO GTPase-dependent signaling pathways, we followed four inclusion criteria: (i) genes encoding the 23 RHO GTPases; (ii) genes encoding proteins involved in the regulation of the RHO GTPase cycle; (iii) genes encoding proteins that have been described as either proximal or distal effectors in previous publications; and (iv) genes encoding proteins that have been described in the proximal interactome of RHO GTPases (Bagci et al., 2020).

### Somatic mutation analyses

TCGA mutation annotation format (MAF) files generated by MuTect2 variant caller pipeline (Benjamin et al., 2019) were downloaded from the Genomic Data Commons (GDC) database using the TCGAbiolinks R package (Colaprico et al., 2016). The TCGA cohorts analyzed were those indicated in Supplementary Table S2. Then, we calculated the mutational burden in samples using the maftools R package (Mayakonda et al., 2018). Samples with the number of mutations 4.5-times higher than the average number of somatic mutations found in the respective analyzed cohort were classified as “hypermutated” and removed from the analysis. From filtered MAF files, we calculated RHO GTPase pathway mutational load and prevalence, along with the background distributions of genes’ mutational load and prevalence in each cohort. To that end, we applied a random sampling approach to create 1,000 lists with the same number of genes as the list of RHO pathway genes (484). These new lists are similar in terms of gene length distribution to the list of RHO pathway genes. This was done to avoid biases in our results from the fact that longer genes often present larger mutational loads in cancer. The normality of each background distribution was assessed using the fitdistrplus R package (https://cran.r-project.org/web/packages/fitdistrplus/index.html). The RHO GTPase pathway and background distribution values were then transformed into Z-scores. Comparisons were finally performed using the Poisson test. *p-*values were further adjusted using the FDR method. A significant threshold was set on an FDR-adjusted *p* < 0.05.

### Analysis of putative oncogenic mutations in RHO pathway genes

We applied two bioinformatics methods to the filtered MAF files. First, we measured the positive selection signals on RHO pathway gene mutations using the dNdScv R package (Martincorena et al., 2017). Genes associated with a qglobal value of <0.01 were considered to harbor mutations under significant positive selection. Genes’ qmiss_cv and qtrunc_cv values were then used, respectively, to differentiate whether missense or truncating mutations were significantly selected on them. Second, we applied the OncodriveCLUSTL algorithm (Arnedo-Pac et al., 2019) to identify clustered mutations (“mutational spots”) on RHO pathway genes. This method was run in each cohort with the following parameters: “smooth windows” of 11, a “simulation window” of 31, and a “mutation cluster cutoff” of 2, 1,000 simulations, and the simulation mode as “mutation_centered.” Finally, we calculated the proportion of a gene’s missense and truncating mutations harbored at these hotspots.

### Differential expression analyses on TCGA datasets

TCGA RNAseq raw counts data derived from the Illumina HiSeq platform were downloaded from the GDC server, processed, and normalized using the TCGAbiolinks R package (Colaprico et al., 2016). First, we performed an array–array intensity correlation (AAIC) analysis to remove outlier samples with an associated whole-transcriptome Pearson correlation value lower than 0.6. Then, genes’ average expression was calculated, and those within the lowest expressed quartile were filtered out. Finally, the resulting matrix of counts was further normalized following the EDAseq R package pipeline (Risso et al., 2011) in two steps: (i) a “within-lane” normalization step to reduce counts’ dependence on genes’ GC content and (ii) a “between-lane” normalization step to reduce their dependence on inter-sample sources of variation. Once normalized, we applied the edgeR differential expression analysis (DEA) pipeline (Mccarthy et al., 2012), which was integrated in the TCGAbiolinks R package. Genes with an associated |log_2_(FC)| value >1 and an FDR-adjusted *p < 0.01* were considered differentially expressed.

### Integration of the somatic copy number and mRNA expression data

To carry out these analyses, we developed a new R-based method compiled in the CiberAMP R package (Caloto et al., 2022). In brief, the data of somatic copy number variations of RHO pathway genes were downloaded from the Broad’s Institute Firebrowse using the TCGAbiolinks R package. We acquired the “thresholded-by-gene” file from the latest GISTIC2.0 run from each cohort (Mermel et al., 2011). Then, tumor samples were classified according to the RHO pathway gene genotype as amplified, deleted, or diploid. We separately analyzed deep and shallow copy number altered samples to associate them with the transcriptional deregulation of RHO pathway genes in each TCGA cohort. Genes associated with a |log_2_(FC)| value >1 and an FDR-adjusted *p* < 0.01 were considered differentially expressed.

Next, we calculated the co-occurrence between the somatic copy number variations of RHO pathway genes and known oncogenes in each cohort. To that end, GISTIC2.0 “thresholded-by-gene” file information (Mermel et al., 2011) was encoded into two binary matrices. On the one hand, in the first matrix, values of 1 encode a deep copy number variation (amplification or deletion) of a given gene in each tumor sample, and values of 0 encode a diploid state. On the other hand, in the second matrix, values of 1 encode a shallow copy number variation (amplification or deletion) of a given gene in each sample, and values of 0 encode a diploid state. Then, we calculated which genes are significantly co-amplified or co-deleted in each cohort. To that end, for every pair of genes within each matrix, we calculated the co-occurrence *p* between their copy number variations using Fisher’s exact test. Two genes were considered significantly co-amplified or co-deleted when associated with an adjusted *p-*value <0.05.

### Gene set enrichment analyses

For these analyses, we followed the steps described in Schaub et al. (2018). In brief, we computed pairwise Spearman correlation coefficients between the pan-cancer up- and downregulated RHO GEFs and RHO GAPs with specific activity for RAC1 and RHOA GTPases and every other transcript in each cohort. The ranked gene list, ordered by decreasing order of Spearman coefficients, was used as input to the gseGO function of the clusterProfiler R package (Wu et al., 2021) to evaluate gene enrichment in biological processes using 10,000 permutations, a maximum gene set size of 800, and a *p* cutoff of 0.01. Finally, we selected the top 100 correlated biological processes from each cohort according to their associated normalized enrichment score (NES).

### Genetic dependence of cancer cell lines on RHO GTPase pathway genes

A total of 1,032 tumor cell lines’ genetic dependency scores from CRISPR/Cas9 genome-wide screens (DepMap 21Q3 Public + Score, Chronos score) and their annotated somatic mutations were downloaded from the Dependency Map (DepMap) database (Pacini et al., 2021; Tsherniak et al., 2017). We considered all those genes associated with a Chronos score < −0.5 in more than 10% of the analyzed tumor cell lines as pan-cancer essential. Then, we compared gene-associated Chronos scores between tumor cell lineages. To that end, we classified cell lines according to their tumor of origin. For each resulting subset of cell lines, we calculated their median Chronos score. This score was then compared to the median Chronos score of all the remaining cell lines using parametric (Student’s *t* test) or non-parametric (Welch’s test) statistical tests. The resulting *p-*values were further adjusted using the FDR method. A comparison was considered statistically significant when associated with an FDR-adjusted *p* < 0.01.

### Correlation between the expression of RHO pathway gene expression and the cell cycle stages

To find out associations between the RHO pathway genes’ expression and the cell cycle of tumor cells, we selected a dataset from HeLa and U2OS cells that were transcriptionally profiled at different cell cycle stages (Bostrom et al., 2017). Analysis of the distribution of the transcripts for the indicated RHO pathway genes within the cell cycle of those cells was carried out using the TriCom algorithm described in that work.

## Data Availability

The original contributions presented in the study are included in the article/Supplementary Material; further inquiries can be directed to the corresponding author.
