# Supplementary material for: Pan-cancer analyses identify oncogenic drivers, expression signatures, and therapeutic vulnerabilities in RHO GTPase pathway genes
Source: Front Bioinform. 2025 Dec 17;5:1708800. doi: 10.3389/fbinf.2025.1708800 (PMC12753894; doi:10.3389/fbinf.2025.1708800)
Supplement: Supplementary file 1 [file DataSheet2.pdf]

Supplementary Table S1. List of RHO GTPase pathway genes used in this study.

Symbol	Subgroup	Chr	Band	Start	End
CDC42	RHO GTPase	1	p36.12	22025511	22101360
RAC1	RHO GTPase	7	p22.1	6374527	6403967
RAC2	RHO GTPase	22	q13.1	37225270	37244448
RAC3	RHO GTPase	17	q25.3	82031678	82034204
RHOA	RHO GTPase	3	p21.31	49359139	49412998
RHOB	RHO GTPase	2	p24.1	20447074	20449440
RHOBTB1	RHO GTPase	10	q21.2	60869438	61001440
RHOBTB2	RHO GTPase	8	p21.3	22987417	23020199
RHOBTB3	RHO GTPase	5	q15	95713522	95824383
RHOC	RHO GTPase	1	p13.2	112701127	112707434
RHOD	RHO GTPase	11	q13.2	67056847	67072017
RHOF	RHO GTPase	12	q24.31	121777754	121803403
RHOG	RHO GTPase	11	p15.4	3826978	3840959
RHOH	RHO GTPase	4	p14	40191053	40246967
RHOJ	RHO GTPase	14	q23.2	63204114	63293508
RHOQ	RHO GTPase	2	p21	46541806	46584688
RHOT1	RHO GTPase	17	q11.2	32142454	32253374
RHOT2	RHO GTPase	16	p13.3	668105	674174
RHOU	RHO GTPase	1	q42.13	228735479	228746664
RHOV	RHO GTPase	15	q15.1	40872214	40874234
RND1	RHO GTPase	12	q13.12	48857145	48865870
RND2	RHO GTPase	17	q21.31	43025231	43032041
RND3	RHO GTPase	2	q23.3	150468195	150539011

Symbol	Subgroup	Chr	Band	Start	End
ABR	RHO GAP and GEF (DH)	17	p13.3	1003519	1229738
AKAP13	RHO GEF (DH)	15	q25.3	85380571	85749358
ALS2	RHO GEF (DH)	2	q33.1	201700267	201782112
ARHGEF1	RHO GEF (DH)	19	q13.2	41883173	41930150
ARHGEF2	RHO GEF (DH)	1	q22	155946851	156007070
ARHGEF3	RHO GEF (DH)	3	p14.3	56727418	57079329
ARHGEF4	RHO GEF (DH)	2	q21.1	130836914	131047263
ARHGEF5	RHO GEF (DH)	7	q35	144355288	144380632
ARHGEF6	RHO GEF (DH)	X	q26.3	136665547	136780932
ARHGEF7	RHO GEF (DH)	13	q34	111114559	111305737
ARHGEF9	RHO GEF (DH)	X	q11.2	63634967	63809274
ARHGEF10	RHO GEF (DH)	8	p23.3	1823926	1958641
ARHGEF10L	RHO GEF (DH)	1	p36.13	17539698	17697874
ARHGEF11	RHO GEF (DH)	1	q23.1	156934840	157045742
ARHGEF12	RHO GEF (DH)	11	q23.3	120336413	120489937
ARHGEF15	RHO GEF (DH)	17	p13.1	8310241	8322514
ARHGEF16	RHO GEF (DH)	1	p36.32	3454665	3481113
ARHGEF17	RHO GEF (DH)	11	q13.4	73308276	73369388
ARHGEF18	RHO GEF (DH)	19	p13.2	7348937	7472485
ARHGEF19	RHO GEF (DH)	1	p36.13	16197854	16212652
ARHGEF25	RHO GEF (DH)	12	q13.3	57610180	57617245
ARHGEF26	RHO GEF (DH)	3	q25.2	154121003	154257827
ARHGEF28	RHO GEF (DH)	5	q13.2	73626158	73941993
ARHGEF33	RHO GEF (DH)	2	p22.1	38889875	38975454
ARHGEF35	RHO GEF (DH)	7	q35	144186083	144195833
ARHGEF37	RHO GEF (DH)	5	q32	149551947	149634968
ARHGEF38	RHO GEF (DH)	4	q24	105552620	105708093
ARHGEF39	RHO GEF (DH)	9	p13.3	35658875	35675866
ARHGEF40	RHO GEF (DH)	14	q11.2	21070273	21090248
BCR	RHO GEF (DH) and GAP	22	q11.23	23179704	23318037
DNMBP	RHO GEF (DH)	10	q24.2	99875577	100009947
ECT2	RHO GEF (DH)	3	q26.31	172750682	172821474
ECT2L	RHO GEF (DH)	6	q24.1	138795911	138904070
FARP1	RHO GEF (DH)	13	q32.2	98142562	98455176
FARP2	RHO GEF (DH)	2	q37.3	241356285	241494841
FGD1	RHO GEF (DH)	X	p11.22	54445454	54496234
FGD2	RHO GEF (DH)	6	p21.2	37005646	37029069
FGD3	RHO GEF (DH)	9	q22.31	92947523	93036236

Symbol	Subgroup	Chr	Band	Start	End
FGD4	RHO GEF (DH)	12	p11.21	32399558	32646050
FGD5	RHO GEF (DH)	3	p25.1	14810853	14934571
FGD6	RHO GEF (DH)	12	q22	95076749	95217482
ITSN1	RHO GEF (DH)	21	q22.11	33642400	33899861
ITSN2	RHO GEF (DH)	2	p23.3	24202864	24360536
KALRN	RHO GEF (DH)	3	q21.1	124033369	124726325
MCF2	RHO GEF (DH)	X	q27.1	139581770	139708227
MCF2L	RHO GEF (DH)	13	q34	112894378	113099742
MCF2L2	RHO GEF (DH)	3	q27.1	183178041	183428778
NET1	RHO GEF (DH)	10	p15.1	5412557	5459056
NGEF	RHO GEF (DH)	2	q37.1	232878701	233013256
OBSCN	RHO GEF (DH)	1	q42.13	228208044	228378876
PLEKHG1	RHO GEF (DH)	6	q25.1	150599883	150843665
PLEKHG2	RHO GEF (DH)	19	q13.2	39412669	39428415
PLEKHG3	RHO GEF (DH)	14	q23.3	64704102	64750249
PLEKHG4	RHO GEF (DH)	16	q22.1	67277510	67289499
PLEKHG4B	RHO GEF (DH)	5	p15.33	92151	189972
PLEKHG5	RHO GEF (DH)	1	p36.31	6467122	6520074
PLEKHG6	RHO GEF (DH)	12	p13.31	6310436	6328506
PLEKHG7	RHO GEF (DH)	12	q22	92702843	92772455
PREX1	RHO GEF (DH)	20	q13.13	48624252	48827999
PREX2	RHO GEF (DH)	8	q13.2	67952046	68237032
SPATA13	RHO GEF (DH)	13	q12.12	23979805	24307074
TIAM1	RHO GEF (DH)	21	q22.11	31118416	31559977
TIAM2	RHO GEF (DH)	6	q25.2	154832697	155257723
TRIO	RHO GEF (DH)	5	p15.2	14143342	14532128
VAV1	RHO GEF (DH)	19	p13.3	6772708	6857366
VAV2	RHO GEF (DH)	9	q34.2	133761894	133992604
VAV3	RHO GEF (DH)	1	p13.3	107571161	107965180
RASGRF1	DH & CDC25 domains	15	q25.1	78959906	79090780
RASGRF2	DH & CDC25 domains	5	q14.1	80960363	81230162
SOS1	DH & CDC25 domains	2	p22.1	38981549	39124345
SOS2	DH & CDC25 domains	14	q21.3	50117130	50231578
DOCK1	RHO GEF (DOCK)	10	q26.2	126905409	127452517
DOCK2	RHO GEF (DOCK)	5	q35.1	169637268	170083382
DOCK3	RHO GEF (DOCK)	3	p21.2	50674927	51384198
DOCK4	RHO GEF (DOCK)	7	q31.1	111726110	112206407
DOCK5	RHO GEF (DOCK)	8	p21.2	25184689	25418082

Symbol	Subgroup	Chr	Band	Start	End
DOCK6	RHO GEF (DOCK)	19	p13.2	11199295	11262524
DOCK7	RHO GEF (DOCK)	1	p31.3	62454298	62688386
DOCK8	RHO GEF (DOCK)	9	p24.3	214854	465259
DOCK9	RHO GEF (DOCK)	13	q32.3	98793429	99086625
DOCK10	RHO GEF (DOCK)	2	q36.2	224765090	225042468
DOCK11	RHO GEF (DOCK)	X	q24	118495815	118686163
RAP1GDS1	RHO GEF (armadillo)	9	q34.13	131576770	131740076

.../...

Symbol	Subgroup	Chr	Band	Start	End
ABR	RHO GAP and GEF (DH)	17	p13.3	1003519	1229738
ARAP1	RHO GAP domain	11	q13.4	72685069	72793599
ARAP2	RHO GAP domain	4	p14	35948221	36244514
ARAP3	RHO GAP domain	5	q31.3	141653401	141682230
ARHGAP1	RHO GAP domain	11	p11.2	46677080	46700619
ARHGAP4	RHO GAP domain	X	q28	153907367	153934999
ARHGAP5	RHO GAP domain	14	q12	32076114	32159728
ARHGAP6	RHO GAP domain	X	p22.2	11117651	11665920
ARHGAP8	RHO GAP domain	22	q13.31	44752558	44862788
ARHGAP9	RHO GAP domain	12	q13.3	57472264	57488814
ARHGAP10	RHO GAP domain	4	q31.23	147732063	148072776
ARHGAP11A	RHO GAP domain	15	q13.3	32615144	32639941
ARHGAP11B	RHO GAP domain	15	q13.2	30624494	30649529
ARHGAP12	RHO GAP domain	10	p11.22	31805398	31928876
ARHGAP15	RHO GAP domain	2	q22.2	143091362	143768352
ARHGAP17	RHO GAP domain	16	p12.1	24919389	25015666
ARHGAP18	RHO GAP domain	6	q22.33	129576132	129710177
ARHGAP19	RHO GAP domain	10	q24.1	97222173	97292673
ARHGAP20	RHO GAP domain	11	q23.1	110577042	110713189
ARHGAP21	RHO GAP domain	10	p12.1	24583609	24723887
ARHGAP22	RHO GAP domain	10	q11.23	48446036	48656265
ARHGAP23	RHO GAP domain	17	q12	38419280	38512385
ARHGAP24	RHO GAP domain	4	q21.23	85475150	86002668
ARHGAP25	RHO GAP domain	2	p13.3	68679601	68826833
ARHGAP26	RHO GAP domain	5	q31.3	142770377	143229011
ARHGAP27	RHO GAP domain	17	q21.31	45393902	45434421
ARHGAP28	RHO GAP domain	18	p11.31	6729716	6915716
ARHGAP29	RHO GAP domain	1	p22.1	94148988	94275068
ARHGAP30	RHO GAP domain	1	q23.3	161046946	161069970
ARHGAP31	RHO GAP domain	3	q13.32	119294383	119420714
ARHGAP32	RHO GAP domain	11	q24.3	128965060	129279324
ARHGAP33	RHO GAP domain	19	q13.12	35774532	35788822
ARHGAP35	RHO GAP domain	19	q13.32	46860997	47005077
ARHGAP36	RHO GAP domain	X	q26.1	131058346	131089885
ARHGAP39	RHO GAP domain	8	q24.3	144529179	144605816
ARHGAP40	RHO GAP domain	20	q11.23	38601934	38651035
ARHGAP42	RHO GAP domain	11	q22.1	100687288	100993941
ARHGAP44	RHO GAP domain	17	p12	12789498	12991643

Symbol	Subgroup	Chr	Band	Start	End
ARHGAP45	RHO GAP domain	19	p13.3	1065922	1086627
BCR	RHO GAP and GEF (DH)	22	q11.23	23179704	23318037
CHN1	RHO GAP domain	2	q31.1	174798809	175005381
CHN2	RHO GAP domain	7	p14.3	29146569	29514328
DEPDC1	RHO GAP domain	1	p31.3	68474152	68497221
DEPDC1B	RHO GAP domain	5	q12.1	60596912	60700190
DLC1	RHO GAP domain	8	p22	13083361	13604610
FAM13A	RHO GAP domain	4	q22.1	88725955	89111398
FAM13B	RHO GAP domain	5	q31.2	137937960	138051961
GMIP	RHO GAP domain	19	p13.11	19629476	19643657
MYO9A	RHO GAP domain	15	q23	71822291	72118577
MYO9B	RHO GAP domain	19	p13.11	17075777	17214537
OCRL	RHO GAP domain	X	q26.1	129539849	129592561
OPHN1	RHO GAP domain	X	q12	67949349	68433913
PIK3R1	RHO GAP domain	5	q13.1	68215756	68301821
PIK3R2	RHO GAP domain	19	p13.11	18153163	18170532
RACGAP1	RHO GAP domain	12	q13.12	49976923	50033136
RALBP1	RHO GAP domain	18	p11.22	9475009	9538114
RALGAPA1	RHO GAP domain	14	q13.2	35538352	35809304
SH3BP1	RHO GAP domain	22	q13.1	37634654	37656117
SRGAP1	RHO GAP domain	12	q14.2	63844700	64162217
SRGAP2	RHO GAP domain	1	q32.1	206203346	206464436
SRGAP3	RHO GAP domain	3	p25.3	8980591	9363053
STARD13	RHO GAP domain	13	q13.1	33103137	33350630
STARD8	RHO GAP domain	X	q13.1	68647666	68725842
SYDE1	RHO GAP domain	19	p13.12	15107401	15114985
SYDE2	RHO GAP domain	1	p22.3	85156889	85201016
TAGAP	RHO GAP domain	6	q25.3	159034468	159045152
ARFGAP2	ARF & RHO GAP domains	11	p11.2	47164299	47177125
ARFGAP3	ARF & RHO GAP domains	22	q13.2	42796502	42858106

Symbol	Subgroup	Chr	Band	Start	End
ARHGDIA	RHO GDI	17	q25.3	81867721	81871378
ARHGDIB	RHO GDI	12	p12.3	14942031	14961728
ARHGDIG	RHO GDI	16	p13.3	280450	283010

Symbol	Subgroup	Type of protein	Chr	Band	Start	End
CDC42BPA	Kinase interactor	Direct effector*	1	q42.13	226989865	227318502
CDC42BPB	Kinase interactor	Direct effector	14	q32.32	102932380	103057549
CDC42BPG	Kinase interactor	Direct effector	11	q13.1	64823052	64844653
CIT	Kinase interactor	Direct effector	12	q24.23	119685791	119877320
CKB	Kinase interactor	Distal effector	14	q32.33	103519667	103522833
DGKA	Kinase interactor	Direct effector	12	q13.2	55927319	55954027
DGKG	Kinase interactor	Direct effector	3	q27.3	186105668	186362234
DGKQ	Kinase interactor	Direct effector	4	p16.3	958887	986895
DMPK	Kinase interactor	Direct effector	19	q13.32	45769709	45782552
INPP5B	Kinase interactor	Direct effector	1	p34.3	37860697	37947057
LIMK1	Kinase interactor	Distal effector	7	q11.23	74082933	74122525
LIMK2	Kinase interactor	Distal effector	22	q12.2	31212239	31280080
MAP3K1	Kinase interactor	Direct effector	5	q11.2	56815549	56896152
MAP3K2	Kinase interactor	Direct effector	2	q14.3	127298668	127388465
MAP3K3	Kinase interactor	Direct effector	17	q23.3	63622415	63696305
MAP3K4	Kinase interactor	Direct effector	6	q26	160991727	161117385
MAP3K5	Kinase interactor	Direct effector	6	q23.3	136557046	136792477
MAP3K6	Kinase interactor	Direct effector	1	p36.11	27354067	27366961
MAP3K7	Kinase interactor	Direct effector	6	q15	90513573	90587072
MAP3K8	Kinase interactor	Direct effector	10	p11.23	30434021	30461833
MAP3K9	Kinase interactor	Direct effector	14	q24.2	70722526	70809534
MAP3K10	Kinase interactor	Direct effector	19	q13.2	40191426	40215575
MAP3K11	Kinase interactor	Direct effector	11	q13.1	65597756	65615382
MAP3K12	Kinase interactor	Direct effector	12	q13.13	53479669	53500063
MAP3K13	Kinase interactor	Direct effector	3	q27.2	185282941	185489094
MAP3K14	Kinase interactor	Direct effector	17	q21.31	45263119	45317029

Symbol	Subgroup	Type of protein	Chr	Band	Start	End
MAP3K15	Kinase interactor	Direct effector	X	p22.12	19360056	19515508
MAP3K19	Kinase interactor	Direct effector	2	q21.3	134964485	135047468
MAP3K21	Kinase interactor	Direct effector	1	q42.2	233327724	233385148
MYLK	Kinase interactor	Direct effector	3	q21.1	123610049	123884332
MYLK2	Kinase interactor	Direct effector	20	q11.21	31819308	31834689
MYLK3	Kinase interactor	Direct effector	16	q11.2	46702282	46790407
MYLK4	Kinase interactor	Direct effector	6	p25.2	2663629	2750922
PAK1	Kinase interactor	Direct effector	11	q14.1	77322017	77474635
PAK2	Kinase interactor	Direct effector	3	q29	196739857	196832647
PAK3	Kinase interactor	Direct effector	X	q23	110944285	111227361
PAK4	Kinase interactor	Direct effector	19	q13.2	39125770	39182816
PAK5	Kinase interactor	Direct effector	20	p12.2	9518036	9819689
PAK6	Kinase interactor	Direct effector	15	q15.1	40217428	40277487
PIK3CA	Kinase interactor	Direct effector	3	q26.32	179148114	179240093
PIP5K1C	Kinase interactor	Direct effector	19	p13.3	3630183	3700468
PKN1	Kinase interactor	Direct effector	19	p13.12	14433053	14471867
PKN2	Kinase interactor	Direct effector	1	p22.2	88684222	88836255
PKN3	Kinase interactor	Direct effector	9	q34.11	128702503	128720916
PLK1	Kinase interactor	Distal effector	16	p12.2	23677656	23690367
PLK2	Kinase interactor	Distal effector	5	q11.2	58453982	58460139
PLK3	Kinase interactor	Distal effector	1	p34.1	44800377	44805990
PTK2	Kinase interactor	Distal effector	8	q24.3	140657900	141002216
PTK2B	Kinase interactor	Distal effector	8	p21.2	27311482	27459391
ROCK1	Kinase interactor	Direct effector	18	q11.1	20946906	21111813
ROCK2	Kinase interactor	Direct effector	2	p25.1	11179759	11348330

Symbol	Subgroup	Type of protein	Chr	Band	Start	End
RPS6KA1	Kinase interactor	Distal effector	1	p36.11	26529761	26575030
RPS6KA2	Kinase interactor	Distal effector	6	q27	166409364	166906451
RPS6KB1	Kinase interactor	Distal effector	17	q23.1	59893046	59950574
RPS6KB2	Kinase interactor	Distal effector	11	q13.2	67428460	67435401
SLK	Kinase interactor	Direct effector	10	q24.33	103967140	104029233
STK10	Kinase interactor	Distal effector	5	q35.1	172042079	172188224
STK38	Kinase interactor	Distal effector	6	p21.31	36493892	36547479
TAOK1	Kinase interactor	Direct effector	17	q11.2	29390363	29551903
TAOK2	Kinase interactor	Direct effector	16	p11.2	29973868	29992261
TAOK3	Kinase interactor	Direct effector	12	q24.23	118149801	118372907
TNK1	Kinase interactor	Direct effector	17	p13.1	7380534	7389774
TNK2	Kinase interactor	Direct effector	3	q29	195863364	195911945
VRK2	Kinase interactor	Distal effector	2	p16.1	57907629	58159920
AAAS	Non-kinase interactor	Proximal interactor	12	q13.13	53307456	53324864
ABCD3	Non-kinase interactor	Proximal interactor	1	p21.3	94418389	94518666
ABI1	Non-kinase interactor	Proximal interactor	10	p12.1	26746593	26861087
ABI2	Non-kinase interactor	Proximal interactor	2	q33.2	203328280	203447728
ABL2	Non-kinase interactor	Proximal interactor	1	q25.2	179099330	179229684
ACBD5	Non-kinase interactor	Proximal interactor	10	p12.1	27168135	27243046
ACTB	Non-kinase interactor	Direct effector	7	p22.1	5526409	5563902
ACTC1	Non-kinase interactor	Direct effector	15	q14	34790230	34795549
ACTG1	Non-kinase interactor	Direct effector	17	q25.3	81509413	81523847
ACTN1	Non-kinase interactor	Direct effector	14	q24.1	68874128	68979440
ACTR2	Non-kinase interactor	Direct effector	2	p14	65227788	65271253
ACTR3	Non-kinase interactor	Direct effector	2	q14.1	113890063	113962596

Symbol	Subgroup	Type of protein	Chr	Band	Start	End
ACTR3B	Non-kinase interactor	Direct effector	7	q36.1	152759749	152855378
ACTR3C	Non-kinase interactor	Direct effector	7	q36.1	150243916	150323725
ADD3	Non-kinase interactor	Proximal interactor	10	q25.1	109996368	110135565
AKAP12	Non-kinase interactor	Proximal interactor	6	q25.1	151239967	151358559
ALDH3A2	Non-kinase interactor	Proximal interactor	17	p11.2	19648136	19685760
AMIGO2	Non-kinase interactor	Proximal interactor	12	q13.11	47075707	47079959
ANKLE2	Non-kinase interactor	Proximal interactor	12	q24.33	132725503	132761832
ANKRD26	Non-kinase interactor	Proximal interactor	10	p12.1	26973793	27100494
ANLN	Non-kinase interactor	Proximal interactor	7	p14.2	36389821	36453791
ARFIP2	Non-kinase interactor	Proximal interactor	11	p15.4	6474683	6481479
ARL13B	Non-kinase interactor	Proximal interactor	3	q11.1	93980139	94055678
ARMCX3	Non-kinase interactor	Proximal interactor	X	q22.1	101622797	101627843
ARPC1A	Non-kinase interactor	Proximal interactor	7	q22.1	99325898	99366262
ARPC1B	Non-kinase interactor	Proximal interactor	7	q22.1	99374249	99394816
ARPC2	Non-kinase interactor	Proximal interactor	2	q35	218217141	218254356
ARPC3	Non-kinase interactor	Proximal interactor	12	q24.11	110434823	110450422
ARPC4	Non-kinase interactor	Proximal interactor	3	p25.3	9792495	9807101
ARPC5	Non-kinase interactor	Proximal interactor	1	q25.3	183620846	183635783
ARPC5L	Non-kinase interactor	Proximal interactor	9	q33.3	124862130	124877733
ATP6AP1	Non-kinase interactor	Proximal interactor	X	q28	154428633	154436516
BAIAP2	Non-kinase interactor	Proximal interactor	17	q25.3	81035122	81117434
BAIAP2L1	Non-kinase interactor	Proximal interactor	7	q22.1	98291650	98401090
BASP1	Non-kinase interactor	Proximal interactor	5	p15.1	17065598	17276843
BCAP31	Non-kinase interactor	Proximal interactor	X	q28	153700492	153724565
BRK1	Non-kinase interactor	Proximal interactor	3	p25.3	10115675	10127190

Symbol	Subgroup	Type of protein	Chr	Band	Start	End
C1QBP	Non-kinase interactor	Proximal interactor	17	p13.2	5432777	5448830
CAPZB	Non-kinase interactor	Proximal interactor	1	p36.13	19338775	19485539
CAV1	Non-kinase interactor	Proximal interactor	7	q31.2	116524994	116561179
CAVIN1	Non-kinase interactor	Proximal interactor	17	q21.2	42402449	42423256
CCDC115	Non-kinase interactor	Proximal interactor	2	q21.1	130337933	130342699
CCDC187	Non-kinase interactor	Proximal interactor	9	q34.3	136249973	136306901
CCDC88A	Non-kinase interactor	Proximal interactor	2	p16.1	55287842	55419895
CCP110	Non-kinase interactor	Proximal interactor	16	p12.3	19523811	19553408
CCT2	Non-kinase interactor	Proximal interactor	12	q15	69585426	69601570
CCT6A	Non-kinase interactor	Proximal interactor	7	p11.2	56051685	56063989
CCT7	Non-kinase interactor	Proximal interactor	2	p13.2	73233420	73253021
CDC42EP1	Non-kinase interactor	Proximal interactor	22	q13.1	37560480	37569405
CDC42EP2	Non-kinase interactor	Proximal interactor	11	q13.1	65314866	65322417
CDC42EP3	Non-kinase interactor	Proximal interactor	2	p22.2	37641882	37738468
CDC42EP4	Non-kinase interactor	Proximal interactor	17	q25.1	73283624	73312005
CDC42EP5	Non-kinase interactor	Proximal interactor	19	q13.42	54465026	54473296
CDC42SE1	Non-kinase interactor	Proximal interactor	1	q21.3	151050971	151070325
CDC42SE2	Non-kinase interactor	Proximal interactor	5	q31.1	131245493	131398447
CEP97	Non-kinase interactor	Proximal interactor	3	q12.3	101724593	101770562
CKAP4	Non-kinase interactor	Proximal interactor	12	q23.3	106237881	106304279
CLTC	Non-kinase interactor	Proximal interactor	17	q23.1	59619689	59696956
COPS2	Non-kinase interactor	Proximal interactor	15	q21.1	49106068	49155661
COPS4	Non-kinase interactor	Proximal interactor	4	q21.22	83034447	83075818
CPD	Non-kinase interactor	Proximal interactor	17	q11.2	30378927	30469989
CPNE2	Non-kinase interactor	Proximal interactor	16	q13	57092583	57148369
CPSF7	Non-kinase interactor	Proximal interactor	11	q12.2	61402641	61430031

Symbol	Subgroup	Type of protein	Chr	Band	Start	End
CYFIP1	Non-kinase interactor	Proximal interactor	15	q11.2	22867052	22981063
DBN1	Non-kinase interactor	Proximal interactor	5	q35.3	177456608	177474401
DBT	Non-kinase interactor	Proximal interactor	1	p21.2	100186919	100249834
DDRGK1	Non-kinase interactor	Proximal interactor	20	p13	3190350	3204685
DDX39B	Non-kinase interactor	Proximal interactor	6	p21.33	31530219	31542448
DDX4	Non-kinase interactor	Proximal interactor	5	q11.2	55738017	55817157
DIAPH1	Non-kinase interactor	Direct effector	5	q31.3	141515016	141619055
DIAPH2	Non-kinase interactor	Direct effector	X	q21.33	96684663	97604997
DIAPH3	Non-kinase interactor	Direct effector	13	q21.2	59665583	60163928
DLG5	Non-kinase interactor	Proximal interactor	10	q22.3	77790791	77926755
DSG1	Non-kinase interactor	Proximal interactor	18	q12.1	31318160	31359246
DSG2	Non-kinase interactor	Proximal interactor	18	q12.1	31498177	31549008
DSP	Non-kinase interactor	Proximal interactor	6	p24.3	7541617	7586714
DST	Non-kinase interactor	Proximal interactor	6	p12.1	56457987	56954830
EFHD2	Non-kinase interactor	Proximal interactor	1	p36.21	15409888	15430339
ELMO2	Non-kinase interactor	Proximal interactor	20	q13.12	46366050	46432985
EMC3	Non-kinase interactor	Proximal interactor	3	p25.3	9962537	10011116
EMD	Non-kinase interactor	Proximal interactor	X	q28	154379273	154381574
EPSTI1	Non-kinase interactor	Proximal interactor	13	q14.11	42886388	42992271
ERBIN	Non-kinase interactor	Proximal interactor	5	q12.3	65926556	66082546
ESYT1	Non-kinase interactor	Proximal interactor	12	q13.2	56118250	56144674
FAF2	Non-kinase interactor	Proximal interactor	5	q35.2	176447628	176510074
FAM65A	Non-kinase interactor	Proximal interactor	16	q22.1	67552321	67580961
FAM65B	Non-kinase interactor	Proximal interactor	6	p11.1	24797601	25042238

Symbol	Subgroup	Type of protein	Chr	Band	Start	End
FAM65C	Non-kinase interactor	Proximal interactor	20	q13.13	49202645	49308065
FAM83B	Non-kinase interactor	Proximal interactor	6	p12.1	54846771	54945099
FAM91A1	Non-kinase interactor	Proximal interactor	8	q24.13	123768439	123815452
FAM135A	Non-kinase interactor	Proximal interactor	6	q13	70412941	70561174
FAM169A	Non-kinase interactor	Proximal interactor	5	q13.3	74777574	74866966
FERMT2	Non-kinase interactor	Proximal interactor	14	q22.1	52857268	52952435
FLOT1	Non-kinase interactor	Proximal interactor	6	p21.33	30727709	30742732
FLOT2	Non-kinase interactor	Proximal interactor	17	q11.2	28879335	28897733
FMNL3	Non-kinase interactor	Proximal interactor	12	q13.12	49636499	49708165
FNBP1	Non-kinase interactor	Proximal interactor	9	q34.11	129887187	130043194
FNBP1L	Non-kinase interactor	Proximal interactor	1	p22.1	93448118	93554661
GARRE1	Non-kinase interactor	Proximal interactor	19	q13.11	34745442	34846491
GFOD1	Non-kinase interactor	Proximal interactor	6	p23	13357830	13487662
GIT1	Non-kinse interactor	Proximal interactor	17	q11.2	29573475	29594054
GIT2	Non-kinse interactor	Proximal interactor	12	q24.11	109929802	109996389
GJA1	Non-kinase interactor	Proximal interactor	6	q22.31	121435595	121449727
GOLGA3	Non-kinase interactor	Proximal interactor	12	q24.33	132768914	132829078
GOLGA8R	Non-kinase interactor	Proximal interactor	15	q13.2	30403740	30414162
GPS1	Non-kinase interactor	Proximal interactor	17	q25.3	82050691	82057470
HGS	Non-kinase interactor	Proximal interactor	17	q25.3	81683326	81703138
HINT2	Non-kinase interactor	Proximal interactor	9	p13.3	35812960	35815354
HMOX2	Non-kinase interactor	Proximal interactor	16	p13.3	4474690	4510347
HNRNPC	Non-kinase interactor	Proximal interactor	14	q11.2	21209136	21269494
HSPE1	Non-kinase interactor	Proximal interactor	2	q33.1	197500140	197503449
IQGAP1	Non-kinase interactor	Proximal interactor	15	q26.1	90388242	90502239
IQGAP2	Non-kinase interactor	Proximal interactor	5	q13.3	76403285	76708132

Symbol	Subgroup	Type of protein	Chr	Band	Start	End
IQGAP3	Non-kinase interactor	Proximal interactor	1	q22	156525405	156572604
ITGB1	Non-kinase interactor	Proximal interactor	10	p11.22	32887273	33005792
JMY	Non-kinase interactor	Proximal interactor	5	q14.1	79236131	79327211
JUP	Non-kinase interactor	Proximal interactor	17	q21.2	41754604	41786931
KCTD3	Non-kinase interactor	Proximal interactor	1	q41	215567304	215621807
KIDINS220	Non-kinase interactor	Proximal interactor	2	p25.1	8721081	8837630
KIF14	Non-kinase interactor	Proximal interactor	1	q32.1	200551497	200620751
KTN1	Non-kinase interactor	Proximal interactor	14	q22.3	55559072	55701526
LAMTOR1	Non-kinase interactor	Proximal interactor	11	q13.4	72085895	72103297
LBR	Non-kinase interactor	Proximal interactor	1	q42.12	225401502	225428925
LEMD3	Non-kinase interactor	Proximal interactor	12	q14.3	65169583	65248355
LETM1	Non-kinase interactor	Proximal interactor	4	p16.3	1811479	1856156
LMAN1	Non-kinase interactor	Proximal interactor	18	q21.32	59327823	59359265
LMNB1	Non-kinase interactor	Proximal interactor	5	q23.2	126776623	126837020
LRRC1	Non-kinase interactor	Proximal interactor	6	p12.1	53794497	53924125
MACO1	Non-kinase interactor	Proximal interactor	1	p36.11	25430858	25500209
MCAM	Non-kinase interactor	Proximal interactor	11	q23.3	119308529	119321521
MOSPD2	Non-kinase interactor	Proximal interactor	X	p22.2	14873421	14922327
MPP7	Non-kinase interactor	Proximal interactor	10	p12.1	28050993	28334486
MPRIP	Non-kinase interactor	Proximal interactor	17	p11.2	17042457	17217679
MTMR1	Non-kinase interactor	Proximal interactor	X	q28	150692971	150765108
MTR	Non-kinase interactor	Proximal interactor	1	q43	236795260	236921278
MUC13	Non-kinase interactor	Proximal interactor	3	q21.2	124905442	124953819
MYH11	Non-kinase interactor	Proximal interactor	16	p13.11	15703135	15857028
MYL12B	Non-kinase interactor	Proximal interactor	18	p11.31	3261479	3278461
MYO6	Non-kinase interactor	Proximal interactor	6	q14.1	75749192	75919537

Symbol	Subgroup	Type of protein	Chr	Band	Start	End
NAP1L1	Non-kinase interactor	Proximal interactor	12	q21.2	76036585	76084735
NCF2	Non-kinase interactor	Proximal interactor	1	q25.3	183555562	183590876
NCK1	Non-kinase interactor	Proximal interactor	3	q22.3	136862208	136951606
NCK2	Non-kinase interactor	Proximal interactor	2	q12.2	105744912	105894274
NCKAP1	Non-kinase interactor	Proximal interactor	2	q32.1	182909115	183038858
NDUFA5	Non-kinase interactor	Proximal interactor	7	q31.32	123536997	123557904
NDUFS3	Non-kinase interactor	Proximal interactor	11	p11.2	47565336	47584562
NHS	Non-kinase interactor	Proximal interactor	X	p22.2	17375200	17735994
NIPSNAP2	Non-kinase interactor	Proximal interactor	7	p11.2	55951793	56000181
NISCH	Non-kinase interactor	Proximal interactor	3	p21.1	52455118	52493068
NOC2L	Non-kinase interactor	Proximal interactor	1	p36.33	944203	959309
NSFL1C	Non-kinase interactor	Proximal interactor	20	p13	1442162	1473842
NUDC	Non-kinase interactor	Proximal interactor	1	p36.11	26900238	26946871
OSBPL11	Non-kinase interactor	Proximal interactor	3	q21.2	125528858	125595497
PAK1IP1	Non-kinase interactor	Proximal interactor	6	p24.2	10694972	10709782
PCDH7	Non-kinase interactor	Proximal interactor	4	p15.1	30720415	31146805
PEAK1	Non-kinase interactor	Proximal interactor	15	q24.3	77100656	77420144
PGRMC2	Non-kinase interactor	Proximal interactor	4	q28.2	128269237	128288829
PHIP	Non-kinase interactor	Proximal interactor	6	q14.1	78934419	79078254
PKP4	Non-kinase interactor	Proximal interactor	2	q24.1	158456952	158682879
PLD1	Non-kinase interactor	Proximal interactor	3	q26.31	171600404	171810950
PLD2	Non-kinase interactor	Proximal interactor	17	p13.2	4807152	4823434
POTEE	Non-kinase interactor	Proximal interactor	2	q21.1	131209536	131265278
PTPN13	Non-kinase interactor	Proximal interactor	4	q21.3	86594315	86815171
RAB7A	Non-kinase interactor	Proximal interactor	3	q21.3	128693669	128825942
RASAL2	Non-kinase interactor	Proximal interactor	1	q25.2	178094104	178484147

Symbol	Subgroup	Type of protein	Chr	Band	Start	End
RBBP6	Non-kinase interactor	Proximal interactor	16	p12.1	24537693	24572863
RBMX	Non-kinase interactor	Proximal interactor	X	q26.3	136848004	136880764
RHPN1	Non-kinase interactor	Proximal interactor	8	q24.3	143368876	143384221
RHPN2	Non-kinase interactor	Proximal interactor	19	q13.11	32978592	33064888
RNF20	Non-kinase interactor	Proximal interactor	9	q31.1	101533853	101563344
RRAS2	Non-kinase interactor	Proximal interactor	11	p15.2	14277922	14364506
RTKN	Non-kinase interactor	Proximal interactor	2	p13.1	74425835	74442422
RTKN2	Non-kinase interactor	Proximal interactor	10	q21.2	62183035	62268844
SCFD1	Non-kinase interactor	Proximal interactor	14	q12	30622291	30737694
SCRIB	Non-kinase interactor	Proximal interactor	8	q24.3	143790920	143815773
SEMA4F	Non-kinase interactor	Proximal interactor	2	p13.1	74654228	74683853
SENP1	Non-kinase interactor	Proximal interactor	12	q13.11	48042897	48106079
SH3PXD2A	Non-kinase interactor	Proximal interactor	10	q24.33	103594027	103855543
SH3RF1	Non-kinase interactor	Proximal interactor	4	q33	169094259	169270956
SH3RF2	Non-kinase interactor	Proximal interactor	5	q32	145936578	146081791
SH3RF3	Non-kinase interactor	Proximal interactor	2	q13	109129205	109504634
SHKBP1	Non-kinase interactor	Proximal interactor	19	q13.2	40576853	40591399
SHMT2	Non-kinase interactor	Proximal interactor	12	q13.3	57229573	57234935
SLC1A5	Non-kinase interactor	Proximal interactor	19	q13.32	46774883	46788594
SLC4A7	Non-kinase interactor	Proximal interactor	3	p24.1	27372721	27484420
SLITRK3	Non-kinase interactor	Proximal interactor	3	q26.1	165186720	165197109
SLITRK5	Non-kinase interactor	Proximal interactor	13	q31.2	87671371	87696272
SNAP23	Non-kinase interactor	Proximal interactor	15	q15.1	42491233	42545356
SOWAHC	Non-kinase interactor	Proximal interactor	2	q13	109614364	109618990
SOX9	Non-kinase interactor	Proximal interactor	17	q24.3	72121020	72126416
SPEN	Non-kinase interactor	Proximal interactor	1	p36.21	15836095	15940456

Symbol	Subgroup	Type of protein	Chr	Band	Start	End
SPTAN1	Non-kinase interactor	Proximal interactor	9	q34.11	128552558	128633662
SPTBN1	Non-kinase interactor	Proximal interactor	2	p16.2	54456317	54671446
SRA1	Non-kinase interactor	proximal interactor	5	q31.3	140537340	140557677
SRRM1	Non-kinase interactor	proximal interactor	1	p36.11	24631716	24673281
STAM	Non-kinase interactor	proximal interactor	10	p12.33	17644151	17716824
STAM2	Non-kinase interactor	proximal interactor	2	q23.3	152116801	152175763
STBD1	Non-kinase interactor	proximal interactor	4	q21.1	76306733	76311130
STEAP3	Non-kinase interactor	proximal interactor	2	q14.2	119223831	119265652
STOM	Non-kinase interactor	proximal interactor	9	q33.2	121338987	121370304
STX5	Non-kinase interactor	proximal interactor	11	q12.3	62806860	62832051
SWAP70	Non-kinase interactor	proximal interactor	11	p15.4	9664077	9752993
TEX2	Non-kinase interactor	proximal interactor	17	q23.3	64147227	64263260
TFRC	Non-kinase interactor	proximal interactor	3	q29	196027183	196082096
TJP2	Non-kinase interactor	proximal interactor	9	q21.11	69121264	69274615
TMEM59	Non-kinase interactor	proximal interactor	1	p32.3	54026681	54053504
TMEM87A	Non-kinase interactor	proximal interactor	15	q15.1	42210447	42273584
TMOD3	Non-kinase interactor	proximal interactor	15	q21.2	51829628	51947295
TMPO	Non-kinase interactor	proximal interactor	12	q23.1	98515579	98550351
TOR1AIP1	Non-kinase interactor	proximal interactor	1	q25.2	179882042	179920077
TPM3	Non-kinase interactor	proximal interactor	1	q21.3	154155304	154194648
TPM4	Non-kinase interactor	proximal interactor	19	p13.12	16067021	16103002
TRA2B	Non-kinase interactor	proximal interactor	3	q27.2	185914558	185938103
TRIOBP	Non-kinase interactor	proximal interactor	22	q13.1	37697048	37776556
TRIP10	Non-kinase interactor	proximal interactor	19	p13.3	6737925	6751530
TUBA1B	Non-kinase interactor	proximal interactor	12	q13.12	49127782	49131397
TWF1	Non-kinase interactor	proximal interactor	12	q12	43793723	43806328

Symbol	Subgroup	Type of protein	Chr	Band	Start	End
TXNL1	Non-kinase interactor	proximal interactor	18	q21.31	56597209	56651600
UACA	Non-kinase interactor	proximal interactor	15	q23	70654554	70763558
UHRF1BP1L	Non-kinase interactor	Proximal interactor	12	q23.1	100028455	100142874
USP9X	Non-kinase interactor	Proximal interactor	X	p11.4	41085445	41236579
VAMP3	Non-kinase interactor	Proximal interactor	1	p36.23	7771296	7781432
VANGL1	Non-kinase interactor	Proximal interactor	1	p13.1	115641970	115698224
VANGL2	Non-kinase interactor	Proximal interactor	1	q23.2	160400564	160428670
VAPB	Non-kinase interactor	Proximal interactor	20	q13.32	58389229	58451101
VCP	Non-kinase interactor	Proximal interactor	9	p13.3	35053928	35072668
VIM	Non-kinase interactor	Proximal interactor	10	p13	17228241	17237593
WAS	Non-kinase interactor	Proximal interactor	X	p11.23	48676596	48691427
WASF1	Non-kinase interactor	Proximal interactor	6	q21	110099819	110180004
WASF2	Non-kinase interactor	Proximal interactor	1	p36.11	27404230	27490167
WASF3	Non-kinase interactor	Proximal interactor	13	q12.13	26557683	26688948
WASL	Non-kinase interactor	Proximal interactor	7	q31.32	123681943	123749003
WDR11	Non-kinase interactor	Proximal interactor	10	q26.12	120851305	120909524
WDR6	Non-kinase interactor	Proximal interactor	3	p21.31	49007062	49015953
WDR81	Non-kinase interactor	Proximal interactor	17	p13.3	1716523	1738599
WDR91	Non-kinase interactor	Proximal interactor	7	q33	135183839	135211555
WHAMM	Non-kinase interactor	Proximal interactor	15	q25.2	82809628	82836108
WIPF1	Non-kinase interactor	Proximal interactor	2	q31.1	174559572	174682916
WIPF2	Non-kinase interactor	Proximal interactor	17	q21.2	40219304	40284136
WIPF3	Non-kinase interactor	Proximal interactor	7	p14.3	29806486	29917066
WWP2	Non-kinase interactor	Proximal interactor	16	q22.1	69762328	69941741
YKT6	Non-kinase interactor	Proximal interactor	7	p13	44200959	44214294
ZNF512B	Non-kinase interactor	Proximal interactor	20	q13.33	63956704	63969930

* **Direct effector,** downstream element that can physically interact with GTPases; **distal effector,** RHO downstream signaling element that is located further downstream of the proximal effectors; **proximal interactor,** protein belonging to the large-scale interactome of specific RHO GTPases according to proteomics determinations.

TOTAL NUMBER OF GENES/PROTEINS

<table>
<tr><th colspan="2">Type</th><th>Number</th><th>Total</th></tr>
<tr><td colspan="2">RHO GTPases</td><td>23 (including 3 RHOBTBs)</td><td>20</td></tr>
<tr><td colspan="2">RHO GAP domain containing proteins</td><td>70
(including 2 with DH domains)</td><td>69</td></tr>
<tr><td rowspan="3">RHO GEFs or contaning similar structural domains</td><td>DH family members</td><td>75 (including 2 with GAP domains). Total 73 genes</td><td rowspan="3">85</td></tr>
<tr><td>DOCK family members</td><td>11</td></tr>
<tr><td>Armadillo domain</td><td>1</td></tr>
<tr><td colspan="2">RHO GDI</td><td>3</td><td>3</td></tr>
<tr><td colspan="2">Downstream elements or interactors with kinase activity</td><td>64</td><td>64</td></tr>
<tr><td colspan="2">Downstream elements or interactors lacking kinase activity</td><td>243</td><td>243</td></tr>
<tr><td colspan="3">Total</td><td>484</td></tr>
</table>
