# Supplementary material for: Pan-cancer analyses identify oncogenic drivers, expression signatures, and therapeutic vulnerabilities in RHO GTPase pathway genes
Source: Front Bioinform. 2025 Dec 17;5:1708800. doi: 10.3389/fbinf.2025.1708800 (PMC12753894; doi:10.3389/fbinf.2025.1708800)
Supplement: Supplementary file 2 [file DataSheet4.pdf]

Supplementary Table S3. List of RHO GTPase pathway genes with alterations found in cancer

Symbol	Subgroup*	Change**
RAC1	RHO GTPase	PS, HS, es
RHOA	RHO GTPase	PS, HS, es
RHOB	RHO GTPase	PS, HS
RND1	RHO GTPase	HS

Symbol	Subgroup	Change
ARAP2	RHO GAP domain	HS
ARHGAP1	RHO GAP domain	HS
ARHGAP9	RHO GAP domain	HS
ARHGAP29	RHO GAP domain	HS
ARHGAP30	RHO GAP domain	HS
ARHGAP35	RHO GAP domain	PS, HS
ARHGAP39	RHO GAP domain	Amp

Symbol	Subgroup	Change
CHN2	RHO GAP domain	HS
DEPDC1	RHO GAP domain	Amp
DEPDC1B	RHO GAP domain	HS
PIK3R1	RHO GAP domain	PS, HS
PIK3R2	RHO GAP domain	HS
STARD13	RHO GAP domain	HS
ARHGEF15	RHO GEF (DH)	HS
ARHGEF25	RHO GEF (DH)	HS
ECT2	RHO GEF (DH)	Amp, ES, es
FGD5	RHO GEF (DH)	HS
MCF2	RHO GEF (DH)	HS
PLEKHG2	RHO GEF (DH)	HS
PLEKHG4	RHO GEF (DH)	HS

Symbol	Subgroup	Change
PLEKHG4B	RHO GEF (DH)	Amp
PLEKHG7	RHO GEF (DH)	HS
DOCK1	RHO GEF (DOCK)	HS

Symbol	Subgroup	Type of protein	Change
CDC42BPA	Kinase interactor	Direct effector*	HS
MAP3K1	Kinase interactor	Direct effector	PS
MYLK	Kinase interactor	Direct effector	HS
PAK6	Kinase interactor	Direct effector	HS
PIK3CA	Kinase interactor	Direct effector	PS, HS
PKN2	Kinase interactor	Direct effector	HS
PLK1	Kinase interactor	Distal effector	HS, UP, ES
RPS6KB1	Kinase interactor	Distal effector	HS
STK10	Kinase interactor	Distal effector	HS
TNK1	Kinase interactor	Direct effector	HS
ACTB	Non-kinase interactor	Direct effector	PS, HS
ACTC1	Non-kinase interactor	Direct effector	HS

Symbol	Subgroup	Type of protein	Change
ACTR3B	Non-kinase interactor	Direct effector	HS
ANLN	Non-kinase interactor	Proximal interactor	Amp, ES
ARL13B	Non-kinase interactor	Proximal interactor	HS
ARPC1A	Non-kinase interactor	Proximal interactor	HS
CKAP4	Non-kinase interactor	Proximal interactor	HS
DST	Non-kinase interactor	Proximal interactor	HS

Symbol	Subgroup	Type of protein	Change
FAM65C	Non-kinase interactor	Proximal interactor	HS
GFOD1	Non-kinase interactor	Proximal interactor	HS
IQGAP3	Non-kinase interactor	Proximal interactor	HS
ITGB1	Non-kinase interactor	Proximal interactor	HS
KIF14	Non-kinase interactor	Proximal interactor	Amp, ES
MTMR1	Non-kinase interactor	Proximal interactor	HS
MYH11	Non-kinase interactor	Proximal interactor	HS, Del
NHS	Non-kinase interactor	Proximal interactor	HS
OSBPL11	Non-kinase interactor	Proximal interactor	HS
PAK1IP1	Non-kinase interactor	Proximal interactor	HS

Symbol	Subgroup	Type of protein	Change
POTEE	Non-kinase interactor	Proximal interactor	HS
PTPN13	Non-kinase interactor	Proximal interactor	HS
RHPN1	Non-kinase interactor	Proximal interactor	Amp
RRAS2	Non-kinase interactor	Proximal interactor	HS
SH3RF2	Non-kinase interactor	Proximal interactor	HS
SLC4A7	Non-kinase interactor	Proximal interactor	HS
SLITRK3	Non-kinase interactor	Proximal interactor	HS
SOX9	Non-kinase interactor	Proximal interactor	PS, ES
SPTAN1	Non-kinase interactor	Proximal interactor	PS
TFRC	Non-kinase interactor	proximal interactor	Amp, es
TRA2B	Non-kinase interactor	proximal interactor	HS
TXNL1	Non-kinase interactor	proximal interactor	HS
WASL	Non-kinase interactor	Proximal interactor	HS
WIPF1	Non-kinase interactor	Proximal interactor	HS

* **Direct effector,** downstream element that can physically interact with GTPases; **distal effector,** RHO downstream signaling element that is located further downstream of the proximal effectors; **proximal interactor,** protein belonging to the large-scale interactome of specific RHO GTPases according to proteomics determinations.

** **PS,** positively selected mutations; **HS,** hotspot mutations; **Amp,** amplified; **Del,** deleted; **ES,** important for proliferation in a large number of cancer cell lines; **es,** important for proliferation of specific cancer cell lines.
