# Supplementary material for: Pan-cancer analyses identify oncogenic drivers, expression signatures, and therapeutic vulnerabilities in RHO GTPase pathway genes
Source: Front Bioinform. 2025 Dec 17;5:1708800. doi: 10.3389/fbinf.2025.1708800 (PMC12753894; doi:10.3389/fbinf.2025.1708800)
Supplement: Supplementary file 4 [file DataSheet1.pdf]

Supplementary Information for

PAN-CANCER ANALYSES IDENTIFY ONCOGENIC DRIVERS, EXPRESSION SIGNATURES, AND THERAPEUTIC VULNERABILITIES IN RHO GTPase PATHWAY GENES

by

R. Fernández *et al*.

This PDF file includes:

Supplementary Figures 1 to 4 and legends

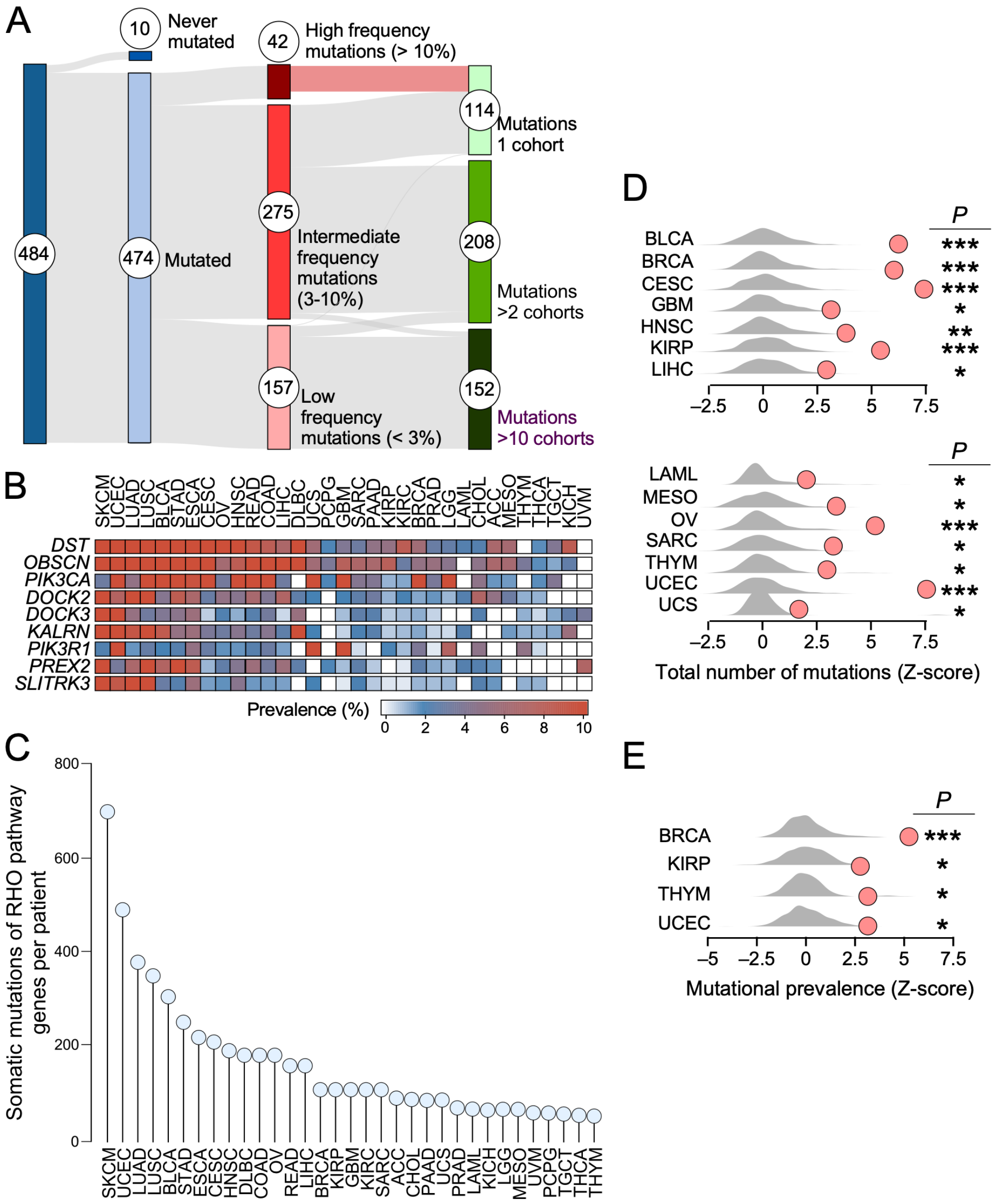

SUPPLEMENTARY FIGURE 1. Somatic mutational landscape of *RHO* pathway genes in TCGA tumors

(A) Sankey diagram with a summary of the information obtained from the analysis of the somatic mutations found on *RHO* pathway genes across TCGA tumors.

(B) Heatmap representing the mutational prevalence of 9 RHO pathway genes mutated in more than one cohort and in at least 10% of the patients. TCGA cancer abbreviations are those shown in Supplementary Table 2.

(C) Somatic mutation burden of RHO pathway genes in indicated tumor types (bottom).

(D and E) Results from the random sampling analysis of the mutational load (D) and prevalence (E) of RHO pathway genes across TCGA cancers comparing RHO pathway genes associated values (red dots) with an expected background distribution (grey areas). *, $P < 0.05$; **, $P < 0.005$; ***, $P < 0.0005$ (Poisson test). Only significant results are represented.

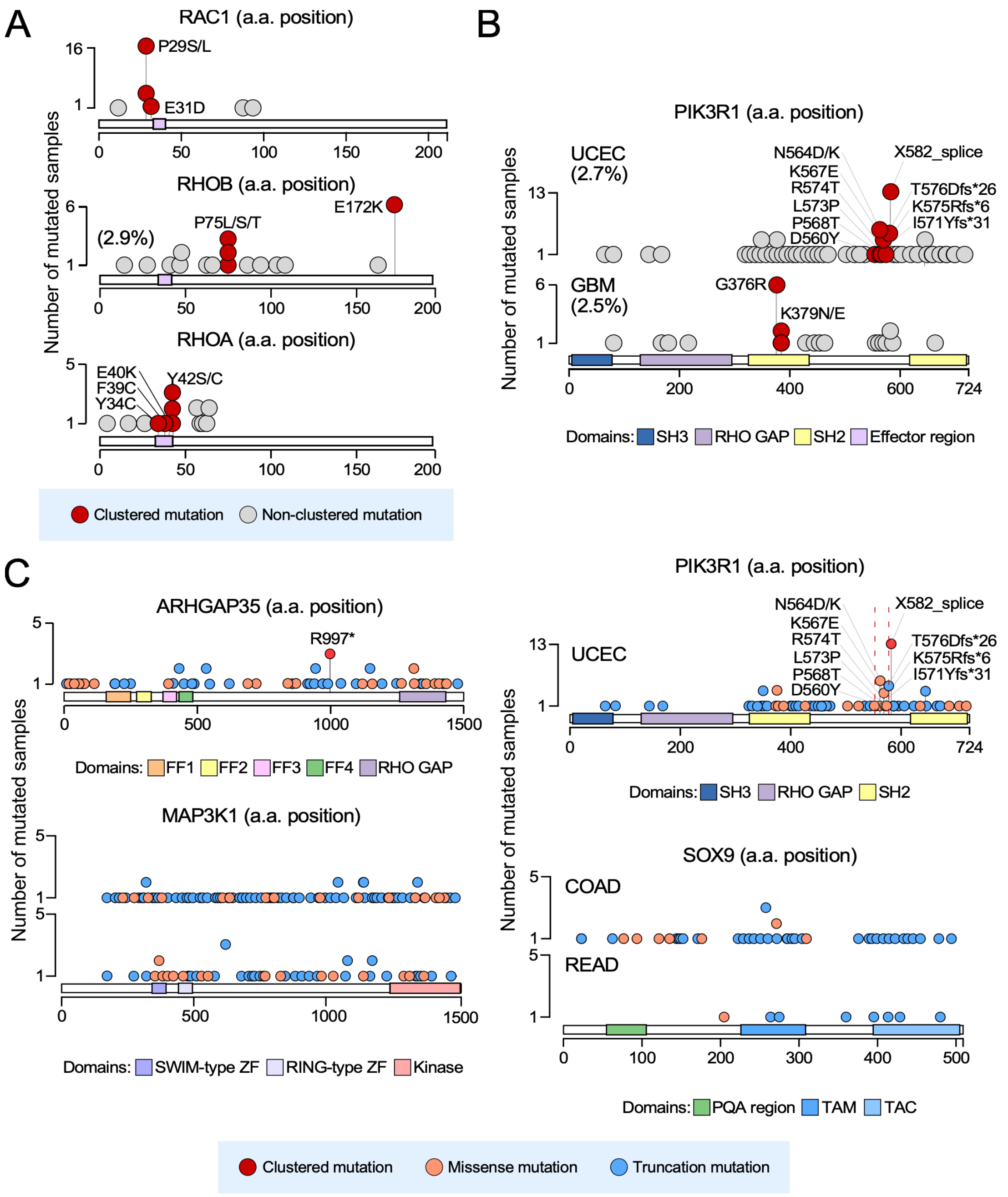

SUPPLEMENTARY FIGURE 2. Examples of hotspot mutations found in RHO pathway genes

(A and B) Needle plots of top prevalent putative oncogenic mutations (red dots) identified among RHO pathway genes in TCGA cancers. a.a, amino acid; *, STOP codon; fs*, frameshift mutation; _splice, mutation affecting splice site.

(C) Needle plots of somatic mutations on RHO pathway genes with truncating alterations that under statistically significant positive selection. Dashed lines depict the limits of the missense-enriched mutational cluster found on *PIK3R1* in UCEC tumors (top panel on the right).

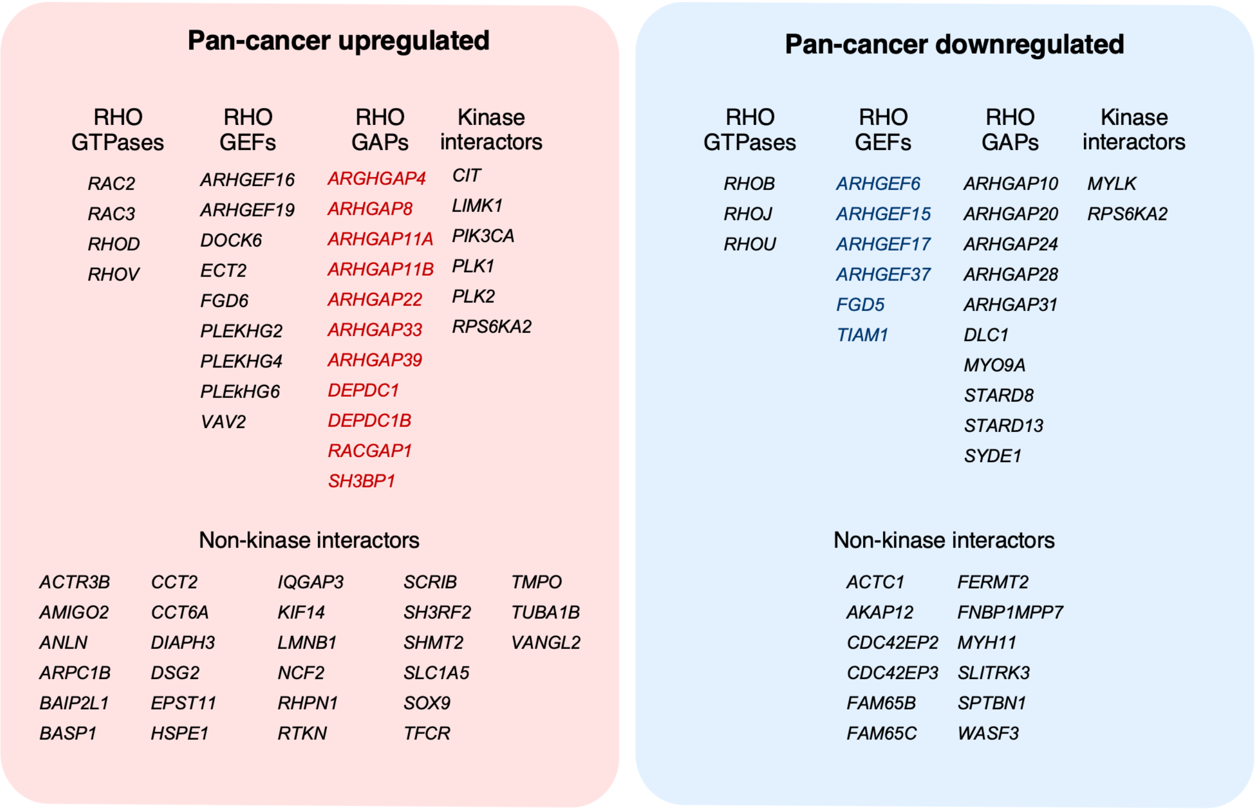

SUPPLEMENTARY FIGURE 3. RHO pathway genes deregulated in pan-cancer data

RHO pathway genes that are upregulated (red box) and downregulated (blue box) in more than 25% of interrogated TCGA tumor cohorts are shown. We also indicate RHO GAPs (red font) and RHO GEFs (blue font) with expression patterns opposite to those expected according to their canonical functions.

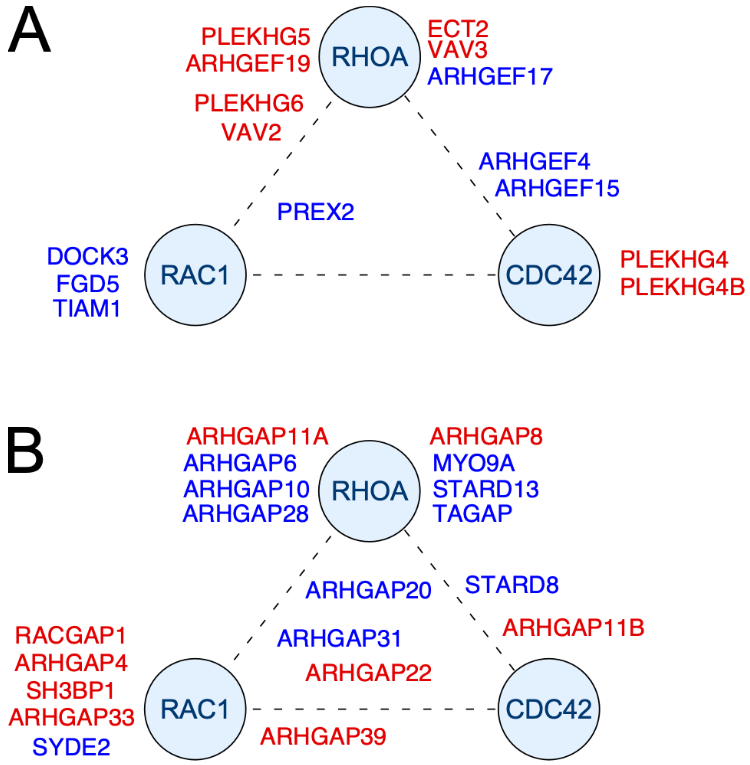

SUPPLEMENTARY FIGURE 4. Functional analysis of RHO GAPs and GEFs with deregulated expression in pan-cancer data

(A and B) Ternary plots showing the catalytic affinity of pan-cancer upregulated (red gene symbols) and downregulated (blue gene symbols) RHO GEFs (A) and RHO GAPs (B) towards the three main RHO subfamily GTPases (CDC42, RAC1, RHOA). The affinity for a given GTPase is represented by the proximity to its corresponding coordinates. See further details in main text.
